# Mammalian Adaptation of an Avian Influenza A Virus Involves Stepwise Changes in NS1

**DOI:** 10.1128/JVI.01875-17

**Published:** 2018-02-12

**Authors:** C. Chauché, A. Nogales, H. Zhu, D. Goldfarb, A. I. Ahmad Shanizza, Q. Gu, C. R. Parrish, L. Martínez-Sobrido, J. F. Marshall, P. R. Murcia

**Affiliations:** a MRC-University of Glasgow Centre for Virus Research, Glasgow, United Kingdom; b Department of Microbiology and Immunology, University of Rochester, Rochester, New York, USA; c Baker Institute of Animal Health, Department of Microbiology and Immunology, College of Veterinary Medicine, Cornell University, Ithaca, New York, USA; d Equine Clinical Sciences Division, Weipers Centre Equine Hospital, School of Veterinary Medicine, University of Glasgow, Glasgow, United Kingdom; St. Jude Children's Research Hospital

**Keywords:** NS1, evolution, influenza, virus-host interactions

## Abstract

Influenza A viruses (IAVs) are common pathogens of birds that occasionally establish endemic infections in mammals. The processes and mechanisms that result in IAV mammalian adaptation are poorly understood. The viral nonstructural 1 (NS1) protein counteracts the interferon (IFN) response, a central component of the host species barrier. We characterized the NS1 proteins of equine influenza virus (EIV), a mammalian IAV lineage of avian origin. We showed that evolutionarily distinct NS1 proteins counteract the IFN response using different and mutually exclusive mechanisms: while the NS1 proteins of early EIVs block general gene expression by binding to cellular polyadenylation-specific factor 30 (CPSF30), NS1 proteins from more evolved EIVs specifically block the induction of IFN-stimulated genes by interfering with the JAK/STAT pathway. These contrasting anti-IFN strategies are associated with two mutations that appeared sequentially and were rapidly selected for during EIV evolution, highlighting the importance of evolutionary processes in immune evasion mechanisms during IAV adaptation.

**IMPORTANCE** Influenza A viruses (IAVs) infect certain avian reservoir species and occasionally transfer to and cause epidemics of infections in some mammalian hosts. However, the processes by which IAVs gain the ability to efficiently infect and transmit in mammals remain unclear. H3N8 equine influenza virus (EIV) is an avian-origin virus that successfully established a new lineage in horses in the early 1960s and is currently circulating worldwide in the equine population. Here, we analyzed the molecular evolution of the virulence factor nonstructural protein 1 (NS1) and show that NS1 proteins from different time periods after EIV emergence counteract the host innate immune response using contrasting strategies, which are associated with two mutations that appeared sequentially during EIV evolution. The results shown here indicate that the interplay between virus evolution and immune evasion plays a key role in IAV mammalian adaptation.

## INTRODUCTION

Influenza A viruses (IAVs) have caused several epizootics in various animal species and four pandemics in humans in the last hundred years. The main natural reservoir of IAVs is wild water birds of various types, but the viruses also circulate in some mammalian host populations, including humans, pigs, horses, and dogs (1). However, most infections of mammals by avian IAVs result in either spillover infections or isolated outbreaks (1, 2), and very few have resulted in the establishment of novel endemic lineages. The reasons underlying the establishment of an avian-origin IAV in a mammalian host are still only partially understood.

In most cases it appears that emerging IAVs have to overcome multiple barriers to infect and become established in a new host. For example, the presence of particular forms or linkages of sialic acids within the respiratory tract can facilitate or impair binding by the viral hemagglutinin (HA) or the activity of the viral neuraminidase (NA) (3, 4). Other incompatibilities between IAVs and host proteins can also account for the species barrier, as it has been recently shown that ANP32A is a host factor that is required for optimal function of the IAV polymerase complex (5). Molecular incompatibilities can sometimes be overcome by adaptive mutations in the virus, and the high mutation rates exhibited by IAVs can facilitate their appearance. The role of mutations in HA in virus-host interaction are among the best characterized (6), but mutations in other viral segments, including those in PB2, nucleoprotein (NP), NA, M, or NS, have also been described (7–9). However, the roles of the latter group in posttransfer adaptation are still incompletely understood.

IAV nonstructural protein 1 (NS1) is encoded by segment eight and possesses two functionally distinct domains: an N-terminal RNA binding domain (RBD) (amino acids 1 to 73) and a C-terminal effector domain (ED) (amino acid 85 to end of protein) separated by a short and flexible linker region (10, 11). The last 25 residues of NS1 are thought to form a disordered and flexible “tail” (10). Molecular interactions with multiple host proteins allow NS1 to perform a remarkable number of activities (12). One of the best-described functions of NS1 is its ability to antagonize the innate immune response, predominantly the production of type I interferons (IFN). The type I IFN response is an essential component of the host barrier that plays a critical role against emerging viruses, as the recipient hosts usually lack preexisting immunity. A variety of pathogen recognition receptors (PRRs) recognize pathogen-associated molecular patterns (PAMPs) and activate a cascade of events that lead to the production and secretion of type I IFN (13). Secreted type I IFN binds to its receptor in both infected and neighboring cells and activates the Janus kinase/signal transducers and activators of transcription (JAK/STAT) pathway, which in turn results in the assembly of a protein complex, referred to as interferon-stimulated gene factor 3 (ISGF3), which is composed of phospho-STAT1, phospho-STAT2, and interferon regulatory factor 9 (IRF9). This complex translocates to the nucleus, binds to DNA regulatory sequences containing IFN-stimulated response elements (ISREs), and stimulates the transcription of hundreds of IFN-stimulated genes (ISGs), including ISG15, myxovirus resistance protein 1 (MX1), and 2′-5′-oligoadenylate synthetase 1/2/3 (OAS1/2/3). As a result, IFN-stimulated cells establish an antiviral state to protect against different viruses, including IAVs (13, 14).

H3N8 equine influenza virus (EIV) is an avian-origin virus lineage that has been circulating in horses since at least 1963 (15, 16) and thus provides a model for the long-term mammalian adaptation of avian-derived IAVs. Phylogenetic studies have shown that several amino acid substitutions occurred within each genomic segment during the evolutionary history of EIV, some of which have been associated with host adaptation in other IAVs (17). However, the role of any of those mutations in EIV adaptation to horses is unclear. We hypothesized that the evolution of NS1 would be part of the process of EIV adaptation to horses. To test this hypothesis, we characterized the NS1 genes of a group of evolutionarily distinct EIVs using a combination of approaches that included experimental infections, reverse genetics, site-directed mutagenesis, phylogenetics, and transcriptomics.

## RESULTS

### Evolutionarily distinct NS1 proteins of the H3N8 EIV lineage exhibit marked functional differences.

To experimentally study the functional evolution of the H3N8 EIV NS1 proteins, we selected 13 H3N8 EIVs that included at least one representative virus per decade since its first isolation in 1963 (Table 1) and cloned the coding sequences of their NS1 proteins into a pCAGGS expression plasmid.

**TABLE 1:** Viruses used in this study

Virus name	Accession no.	Abbreviation
A/equine/Uruguay/1/1963	ACD85423	U/63
A/equine/Miami/1/1963	ABY81497	M/63
A/equine/SaoPaulo/1/1969	ACD85390	SP/69
A/equine/Fontainebleau/1/1979	ACD85401	F/79
A/equine/Sussex/1/1989	ACD97430	S/89
A/equine/Kentucky/1/1991	ACA24672	K/91
A/equine/LaPlata/1995	MF182460	LP/95
A/equine/Kentucky/1995	MF182451	K/95
A/equine/Kentucky/1999	MF182443	K/99
A/equine/Kentucky/5/2002	ABA42429	K/02
A/equine/Newmarket/5/2003	ACI48802	N/03
A/equine/Ohio/1/2003	ABA42431	O/03
A/equine/Mongolia/3/2013	MF182459	M/13

**FIG 1:**
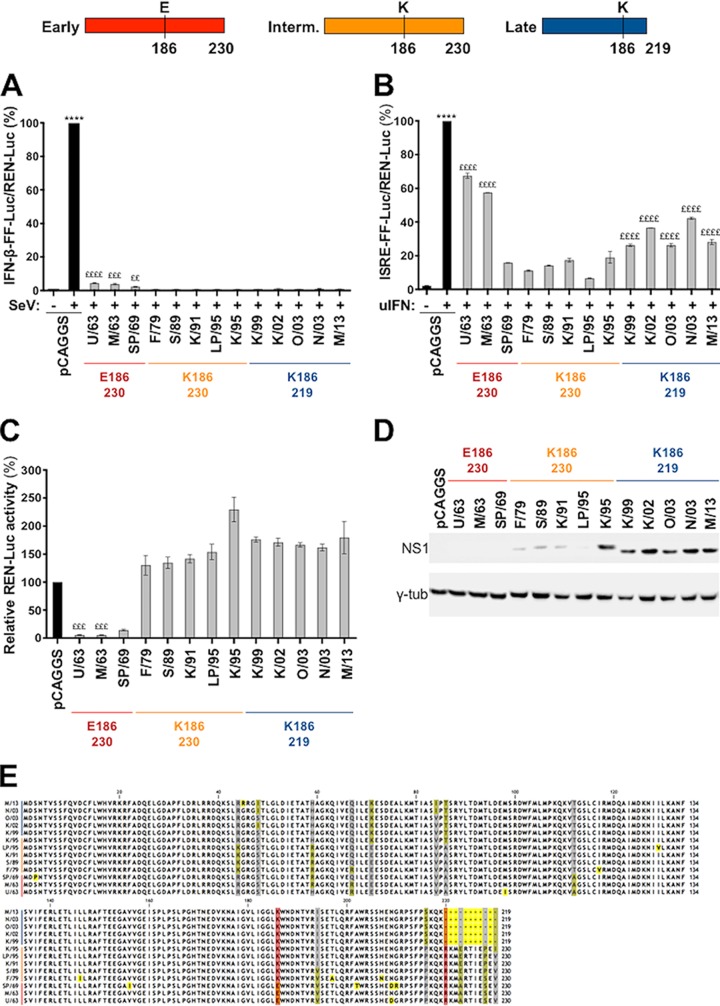
Functional characterization of evolutionarily distinct EIV NS1 proteins. (Top) Schematic representation of three versions of the NS1 protein (early, intermediate, and late) found throughout EIV evolution. Early NS1 proteins, full length (230 amino acids) and harboring E186, were found in EIVs circulating between the 1960s and 1970s; intermediate (Interm.) NS1 proteins, full length with K186, were found in EIVs circulating between the late 1970s and late 1990s; and late NS1 proteins, truncated at the C terminus (219 amino acids) with K186, are found in EIVs isolated since the late 1990s. (A and B) Human 293T cells were transiently cotransfected with a pCAGGS expression plasmid encoding the indicated NS1 proteins (or with empty plasmid), together with either a firefly luciferase IFN-β promoter reporter construct (pIFN-β-FF-Luc) (A) or a plasmid expressing firefly luciferase under the control of a promoter containing an IFN-stimulated regulatory element (pISRE-FF-Luc) (B), as well as with a constitutively active Renilla luciferase expression plasmid (pREN-Luc). At 24 hpt, the cells were either infected with SeV (+) or mock infected (−) (A) or treated with uIFN (+) or mock treated (−) (B) for 18 h. The relative activity of FF-Luc in cells transfected with the pIFN-β-FF-Luc (A) or pISRE-FF-Luc (B) plasmid was determined as the ratio between FF-Luc and REN-Luc in each corresponding sample. The values were normalized to the empty pCAGGS plasmid plus SeV (A) or the empty pCAGGS plasmid plus uIFN (B) (set to 100%). (C) Human 293T cells were transiently cotransfected individually with the indicated NS1 expression plasmids together with pREN-Luc. Total REN-Luc levels were measured 24 h later, and the values were normalized to empty-pCAGGS-transfected cells. (A to C) The bars represent means of the results from three independent experiments, and the error bars represent SEM. Significance was calculated as indicated in Materials and Methods. ****, *P* < 0.0001 for pCAGGS plus SeV or pCAGGS plus uIFN versus all other conditions. ££, *P* < 0.01; ££, *P* < 0.001; £££, *P* < 0.0001 for the indicated NS1 against all other NS1 proteins. (D) The level of expression of the indicated NS1 was analyzed by Western blotting from parallel transfected lysates using a rabbit polyclonal anti-EIV NS1 antibody. (E) Alignment of NS1 amino acid sequences of the 13 EIVs used in this study. Yellow shading, polymorphism between the 13 NS1 proteins; red shading, NS1 amino acid changes investigated in the study; gray shading, other polymorphisms present at high frequency in the EIV population.

As the F2/F3 region of CPSF30 involved in interaction with influenza virus NS1 proteins is conserved between human and equine species (data not shown) and the transfection efficiency of 293T cells is highly superior to that of equine dermal fibroblasts (E-derm cells) (data not shown), we used 293T cells to study the functional evolution of EIV NS1. To compare the abilities of the individual NS1 proteins to limit the production of IFN-β, we cotransfected 293T cells with NS1 expression plasmids or with an empty vector (pCAGGS), together with a plasmid expressing firefly luciferase (FF-Luc) under the IFN-β promoter (pIFN-β-FF-Luc) and with a constitutively active Renilla luciferase (REN-Luc) expression plasmid (pREN-Luc) to normalize for transfection efficacy. At 24 h posttransfection (hpt), the cells were infected with Sendai virus (SeV), a well-described IFN-β inducer. As expected, SeV infection resulted in strong activation of the IFN-β promoter in cells cotransfected with the empty vector (set to 100%) (Fig. 1A) compared to uninfected control cells. However, SeV activation of the IFN-β promoter was blocked in cells transfected with the different EIV NS1-expressing plasmids used in this study (Fig. 1A).

We also compared the abilities of these NS1 proteins to block the induction of ISGs upon IFN treatment. To this end, we cotransfected 293T cells with the NS1 expression plasmids described above, together with a plasmid expressing firefly luciferase under the control of a promoter containing an IFN-stimulated regulatory element (pISRE-FF-Luc), and again used pREN-Luc as an internal control. At 24 hpt, we treated the cells with universal type I IFN (uIFN) (PBL Assay Science; 500 units/well) to stimulate the ISRE-containing promoter, and 18 h later, we measured FF-Luc and REN-Luc activities. The NS1 proteins of EIVs isolated in 1963 (so-called “early”: U/63 and M/63) displayed relatively low repression of the ISRE-containing promoter (Fig. 1B), and the antagonistic property of NS1 over this promoter increased in EIVs that circulated between 1969 and 1995 (SP/69, F/79, S/89, K/91, LP/95, and K/95). Furthermore, the NS1 proteins of EIVs isolated between 1999 and 2013 (so-called “late”: K/99, K/02, O/03, N/03, and M/13) showed variable control of the ISRE-containing promoter, depending on the NS1 protein tested (Fig. 1B).

To assess the abilities of the NS1 proteins to inhibit general gene expression, we performed cotransfections of the individual NS1 expression plasmids, together with pREN-Luc, and measured luciferase activity 24 h later. The NS1 proteins of EIVs that circulated in the 1960s strongly blocked the otherwise constitutively active Renilla luciferase (Fig. 1C). In contrast, the NS1 proteins of EIVs isolated after 1979 did not block general gene expression, as REN-Luc expression was not strongly reduced. In addition, in some cases, NS1 expression was associated with an increase in luciferase expression (Fig. 1C), as described for other NS1 proteins (18). We also examined the expression levels of NS1 by Western blotting in parallel transfected-cell lysates. As expected, the NS1 proteins that strongly blocked general gene expression (i.e., U/63, M/63, and SP/69) could not be detected, probably because of the ability of these NS1 proteins to inhibit their own synthesis (18, 19) (Fig. 1D). On the other hand, NS1 proteins that did not block host gene expression (F/79 to M/13) were easily detected. Interestingly, the level of expression of NS1 proteins of viruses isolated between 1979 and 1995 (F/79 to LP/95) was lower than that of more recent NS1 proteins (K/95 to M/13).

Taken together, these results suggest that the NS1 proteins of the H3N8 EIV lineage maintained strong control over IFN-β production throughout evolution while progressively increasing their control of IFN-stimulated signal transduction. Such control mechanisms are likely independent of the NS1-mediated control of general gene expression, as the NS1 proteins of EIVs that circulated after 1969 lost that ability. These results suggest a direct effect of these NS1 proteins on the IFN signaling pathway.

### Amino acid 186 and the C-terminal tail affect EIV NS1 protein function.

To identify the mutations responsible for the stepwise chronological changes in EIV NS1 function, we first examined a multiple-sequence alignment of the NS1 proteins used in our studies (Fig. 1E). While various amino acid changes were observed, the most evident was an 11-amino-acid truncation in the carboxy (C) terminus that appeared in 1999 (20), as well as the appearance of two amino acid substitutions in the 1970s: E186K and A112T. The E186K substitution lies within the putative CPSF30-binding domain, and notably, this substitution took place at the same time as the loss of repression of general gene expression by EIV NS1 (Fig. 1C).

**FIG 2:**
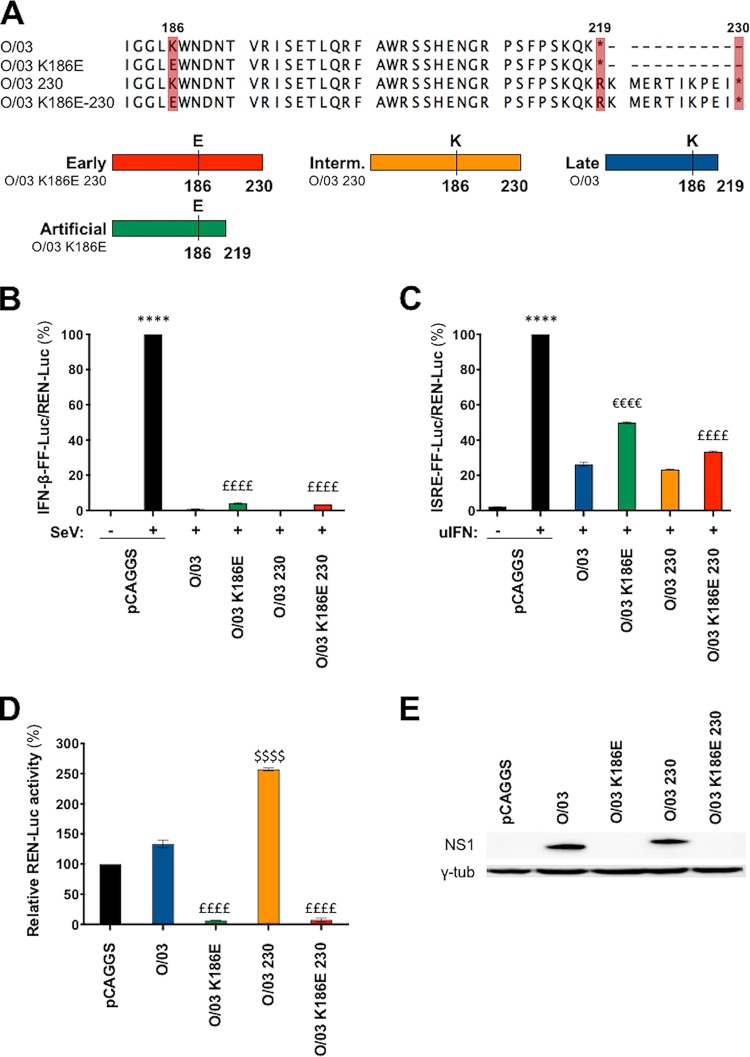
NS1 E186 controls general gene expression. (A) Protein alignment showing the sequence of interest and the introduced mutations in EIV NS1. Below the alignment is a diagram of the three different NS1 proteins that represent evolutionarily distinct NS1 proteins: early NS1, O/03-230, 230 amino acids long and possessing E186; intermediate NS1, O/03-230, 230 amino acids long and possessing K186; and late NS1, 219 amino acids long and possessing K186. (B to D) IFN-β (B), ISRE (C), and general gene (D) expression reporter assays were done using the indicated NS1 pCAGGS expression constructs as described for Fig. 1. Experiments were performed three times independently, and significance was calculated as described in Materials and Methods. ****, *P* < 0.0001 for pCAGGS plus SeV or pCAGGS plus uIFN versus all other conditions. ££££, significant difference (*P* < 0.0001) between K186E-containing revertant(s), O/03, and O/03-230 revertant; €€€€, significant (*P* < 0.0001) difference between O/03-K186E and all other conditions; $$$$, significant difference (*P* < 0.0001) between O/03 and O/03-230 revertant. The error bars represent SEM. (E) Western blot of lysates from parallel samples performed as described for Fig. 1.

To determine the impacts of the E186K substitution and the C-terminal truncation on NS1 function, we introduced mutations in the NS1 gene of A/equine/Ohio/2003 (O/03), a virus isolated after 40 years of continuous EIV circulation in horses. The NS1 protein of O/03 is 219 amino acids long (naturally truncated) and possesses a lysine at position 186 (Fig. 2A, Late). Three NS1 revertant viruses were tested: a K186E substitution (O/03-K186E), a C-terminal extension of 11 amino acids (O/03-230), and the double change of the extended NS1 and the K186E substitution together (O/03-K186E-230) (Fig. 2A). The O/03-230 NS1 revertant would therefore represent the intermediate (Fig. 2A, Interm.) NS1 proteins of EIVs that circulated between 1979 and 1995 (F/79, S/89, K/91, LP/95, and K/95), while the NS1 double revertant represents early NS1 proteins of EIVs that circulated between 1963 and 1969 (U/63, M/63, and SP/69) (Fig. 2A, Early). To determine the impact of a glutamic acid residue at position 186, we introduced a K186E mutation in the O/03 NS1 (Fig. 2A, Artificial). However, it should be noted that such a revertant has not been detected in nature.

The presence of a glutamic acid at position 186 in O/03-K186E and O/03-K186E-230 was associated with decreased ability to control the IFN-β promoter (Fig. 2B), decreased capacity to control the activity of the ISRE-containing promoter (Fig. 2C), and strong repression of the constitutively expressed promoter compared to O/03 and an O/03-230 revertant (Fig. 2D). Consistent with this result, NS1 proteins harboring E186 were not detectable by Western blotting (Fig. 2E).

**FIG 3:**
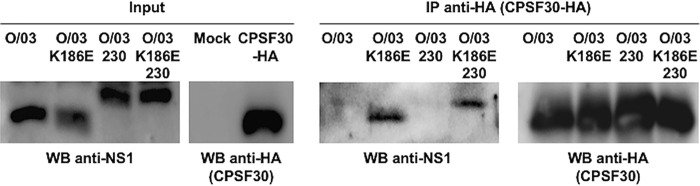
Amino acid 186 is a determinant of NS1 interaction with CPSF30. NS1 variants were synthesized *in vitro*. Human 293T cells were transiently transfected with 2,000 ng of a pCAGGS plasmid expressing an HA-tagged version of the equine CPSF30. At 30 hpt, the cells were lysed, and cleared cell lysates expressing HA-CPSF30 were incubated overnight at 4°C with the *in vitro*-synthesized NS1 proteins and 20 μl of an anti-HA affinity resin. Precipitated proteins were dissociated from the resin and analyzed by Western blotting (WB). Representative blots showing protein expression levels (Input) and coimmunoprecipitation results (IP anti-HA) are shown.

Since NS1 binding to CPFS30 is known to block general gene expression (12), we performed coimmunoprecipitation assays using an equine CPSF30 and NS1 O/03 and revertant proteins expressed *in vitro*. We confirmed that the presence of E186 determines CPSF30 binding, regardless of the length of NS1 (Fig. 3). It should be noted that binding of NS1 to CPSF30 is consistent with the inhibition of NS1 expression upon transfection (Fig. 1D and 2D). This is because transcription from the pCAGGS plasmid is driven by a polymerase II (Pol II)-dependent promoter through binding to CPSF30.

Overall, these results suggested that the NS1 proteins of early EIVs were able to control general gene expression through CPSF30 binding and that the K186E substitution, which arose in NS1 approximately 10 years after emergence of the virus, influenced the control of IFN and ISG induction. Moreover, this substitution seems to have released the block of general gene expression and improved NS1's ability to control IFN-mediated signal transduction.

### Mutations in NS1 acquired throughout evolution affect virus replication and cell-to-cell spread.

**FIG 4:**
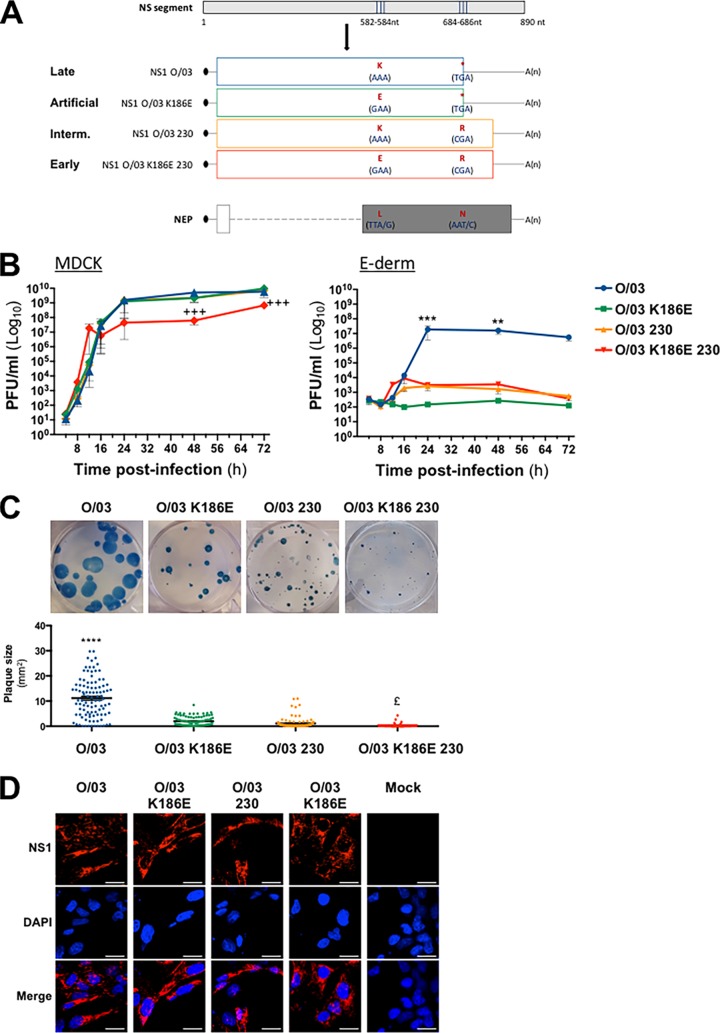
Characterization of EIVs carrying mutations in NS1. (A) Schematic representation of the mutations introduced in the O/03 NS segment at NS1 codon 186 and codon 220 (nucleotides 582 to 584 and 684 to 686 in the O/03 NS segment, respectively) and the resulting amino acid changes in the different NS1 proteins (late, artificial, intermediate, and early). A(n) indicates a poly(A) tail, and an asterisk indicates a stop codon (TGA). The introduced changes did not change the NEP amino acids at positions 28 (TTA/G, coding for leucine) and 68 (AAT/C, coding for asparagine). (B) Growth kinetics of O/03, O/03-K186E, O/03-230, and O/03-K186E-230 viruses in MDCK cells (MOI, 0.01) and E-derm cells (MOI, 0.1). The data points represent virus titers in supernatants at different times postinfection, and the error bars represent SEM. (C) Plaque phenotype of O/03, O/03-K186E, O/03-230, and O/03-K186E-230 in MDCK cells at 48 hpi. The average plaque size for each virus was determined by counting over 100 plaques per condition. Significance was calculated as described in Materials and Methods. +++, *P* < 0.001 for O/03-K186E-230 versus O/03. ****, *P* < 0.0001; ***, *P* < 0.001; **, *P* < 0.01 for O/03 versus the other three viruses. £, *P* < 0.05 for O/03-K186E-230 versus O/03-K186E. (D) Subcellular localization of NS1 in infected equine cells. E-derm cells were infected with O/03, O/03-K186E, O/03-230, and O/03-K186E-230 (MOI, 0.1) for 24 h. NS1 was detected by immunofluorescence as described in Materials and Methods. The experiment was performed three times independently, and representative images of NS1 are shown. Scale bars, 20 μm.

Three individual O/03 revertant viruses were generated and tested for growth in Madin-Darby canine kidney (MDCK) cells: one carrying the K186E substitution (O/03-K186E), another carrying the C terminus extension (O/03-230), and another one carrying both changes (O/03-K186E-230). As indicated in Fig. 4A, none of the introduced changes modified the nuclear export protein (NEP) amino acid sequence. While the O/03-K186E and O/03-230 viruses exhibited growth kinetics similar to those of O/03 (Fig. 4B), the double revertant O/03-K186E-230 displayed a higher replication rate until 12 h postinfection (hpi) but never reached a titer as high as those of the three other viruses over 72 h (Fig. 4B).

In equine cells, the O/03 virus showed a significant advantage over all the revertant viruses after 24 hpi. Indeed, O/03 peaked at a titer 3 to 4 log units higher than those of the revertants between 24 and 72 hpi (Fig. 4B). Interestingly, during the first 16 hpi, no difference in growth kinetics was observed between O/03 and natural revertant viruses (O/03-230 and O/03-K186E-230). More importantly, O/03-K186E-230 displayed the highest replication rate during the first 12 hpi, reaching a titer 2 log units higher than that of any other virus tested. The O/03-K186E revertant, which expressed an NS1 protein that was never isolated in nature, showed strong attenuation compared to O/03 between 16 and 72 hpi.

When we tested the abilities of O/03 and revertant viruses to spread among neighboring cells by examining their plaque phenotypes in MDCK cells, all the revertant viruses displayed significantly smaller plaques than O/03 (Fig. 4C), and the O/03-K186E-230 revertant was the most affected, showing pinpoint plaques. This suggested that NS1 evolution resulted in more efficient cell-to-cell spread.

As NS1 proteins harboring E186 bind CPSF30, we wanted to check if during viral infection NS1 would be present in the same intracellular compartment as CPSF30 (i.e., the nucleus). All the proteins exhibited similar nucleocytoplasmic localization patterns (Fig. 4D), which suggests that the introduced mutations did not affect NS1 subcellular localization.

### NS1 amino acid 186 and the length of the C-terminal tail are key determinants of EIV control of cellular protein synthesis and the establishment of an antiviral state in equine cells.

**FIG 5:**
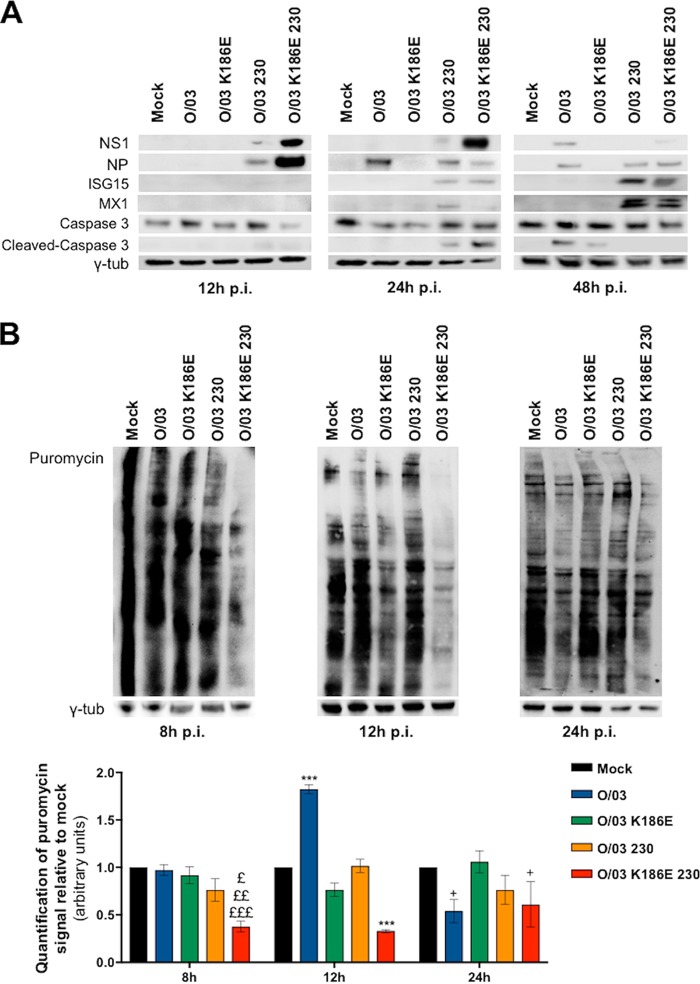
NS1 amino acid 186 and C-terminal tail affect EIV control of general protein production, apoptosis, and response to IFN in equine cells. E-derm cells were infected (MOI, 0.1) with O/03, O/03-K186E, O/03-230, and O/03-K186E-230 viruses. (A) Cells were lysed and immunoblotted for the indicated proteins at the indicated times postinfection (p.i.). (B) To measure protein shutoff upon infection, culture media were replaced with a puromycin-containing suspension and incubated for 1 h. The cells were then lysed and immunoblotted for puromycin at the indicated times, as described in Materials and Methods. Western blot quantifications for puromycin and γ-tubulin signals were determined for three independent experiments and expressed relative to mock-infected cells. Significance was calculated as indicated in Materials and Methods. ***, *P* < 0.001 for the indicated virus versus all other conditions at 12 hpi. £, *P* < 0.05 for O/03-K186E-230 versus 230; ££, *P* < 0.01 for O/03-K186E-230 versus O/03-K186E; £££, *P* < 0.0001 for O/03-K186E-230 versus O/03 and mock infection. +, *P* < 0.05 for the indicated virus versus mock-infected samples at 24 hpi. Gamma-tubulin was used as a loading control. The error bars indicate SEM.

To compare the viruses' abilities to limit the establishment of an antiviral state, we infected equine cells (multiplicity of infection [MOI], 0.1) with each of the viruses and monitored the expression of two ISGs (ISG15 and MX1) by Western blotting at different times postinfection. As expected, ISG15 and MX1 were detected in lysates of cells infected with both the O/03-230 and O/03-K186E-230 revertant viruses, but not O/03 (Fig. 5A), at 24 hpi. We did not detect the proteins in cells infected with O/03-K186E.

**FIG 6:**
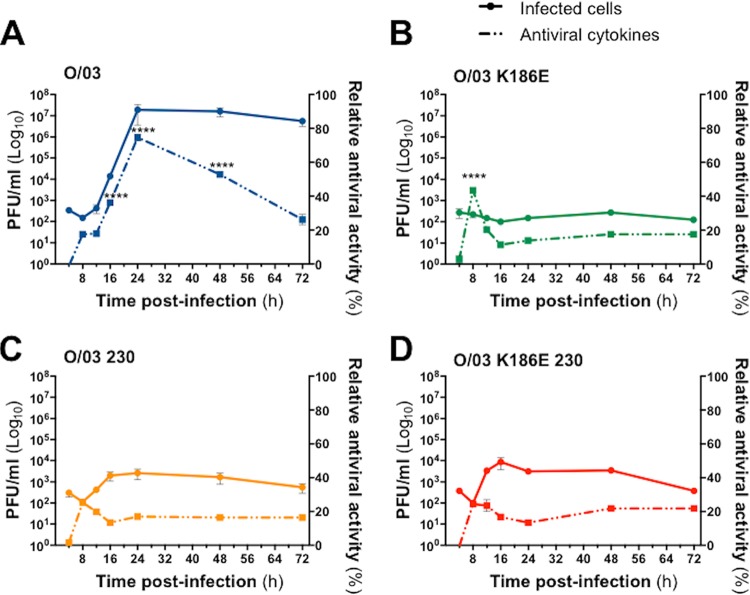
Comparison of growth kinetics and production of antiviral cytokines in equine cells infected with EIV O/03 and NS1 revertant viruses. E-derm cells were infected (MOI, 0.1) for 72 h with O/03 (A), O/03-K186E (B), O/03-230 (C), and O/03-K186E-230 (D). Supernatants were collected, and the cells were fixed at the indicated times postinfection. The total amount of antiviral cytokines produced upon infection was measured using an IFN bioassay, and virus growth kinetics was determined by immunofocus assay. The error bars represent SEM. Significance was calculated as indicated in Materials and Methods. ****, *P* < 0.0001 for the indicated virus at the indicated time postinfection versus the other three viruses at the same time postinfection.

To determine the effects of the NS1 revertants on the synthesis of cellular proteins, we performed puromycin assays at various times postinfection in equine cells (21). Puromycin is incorporated at the C termini of all nascent proteins (22). After cell lysis, immunoblotting against puromycin was performed to allow the detection of nascent proteins of various sizes (appearing as a black smear) that were being produced at the moment of cell lysis. The darker and longer the smear was, the more proteins were being produced at the moment of cell lysis. The double-revertant virus (O/03-K186E-230) induced strong protein shutoff (Fig. 5B), particularly at early times postinfection, and it was associated with high expression levels of NS1 from 8 to 24 hpi (Fig. 5A). As expected, the presence of E186 did not lead to self-inhibition upon infection (in contrast with the self-inhibition of NS1 observed in transfections [Fig. 3]). This is because, during viral infections, the viral polymerase complex drives transcription of NS1, and this process does not require CPSF30. Differences in molecular mechanisms between transfections and infections have also been reported in the literature (18, 19, 23). While this result is consistent with E186 playing an important role in blocking general gene expression via a CPSF30-dependent mechanism, it also suggests that a complementary role of the NS1 C-terminal tail might be necessary to induce strong protein shutoff, as O/03-K186E did not block protein synthesis at any time postinfection (Fig. 5B). However, we cannot rule out the possibility that this lack of protein shutoff was due to a low infection level or low expression of O/03-K186E NS1 protein in equine cells, likely due to strong attenuation of the virus in equine cells (Fig. 6A).

Interestingly, the O/03 virus induced a significant increase in protein synthesis at 12 hpi and low but significant protein shutoff at 24 hpi (Fig. 5B).

As the double-revertant virus (O/03-K186E-230) induced strong protein shutdown and an early antiviral state, we assumed that cellular homeostasis would be compromised and the cells would undergo apoptosis prematurely. To test this hypothesis, we compared the expression levels of caspase 3 (cleaved and total) in O/03 and revertant virus-infected E-derm cells (MOI, 0.1) at different times postinfection (Fig. 5A). As expected, cleavage of caspase 3 was already detectable at 24 hpi in cells infected with the O/03-K186E-230 revertant virus. Interestingly, the O/03-230 revertant virus also induced premature apoptosis, suggesting that the tail of NS1 plays a role in controlling programmed cell death.

### EIV O/03 grows to high levels despite eliciting large amounts of antiviral cytokines and can replicate in IFN-primed equine cells.

To assess whether the IFN system was the limiting factor of O/03 revertant virus replication, we measured the antiviral activities of the supernatants of equine cells infected with the viruses (MOI, 0.1) at different times postinfection using a recombinant vesicular stomatitis virus (rVSV)-green fluorescent protein (GFP)-based bioassay (24) and compared them with the growth kinetics of each virus (Fig. 6). Surprisingly, the highest level of antiviral cytokines was produced upon O/03 infection, peaking at 24 hpi (Fig. 6A). This did not seem to affect viral growth, since the virus rapidly reached a very high titer (10^7^ PFU/ml at 24 hpi), as previously shown (Fig. 4B). The patterns of antiviral-cytokine production compared to viral growth were similar between O/03-230 and O/03-K186E-230 revertant viruses (Fig. 6C and D, respectively), where low levels of antiviral cytokines were associated with limited virus growth. In contrast, cells infected with the O/03-K186E revertant virus exhibited a significantly high peak of antiviral cytokines at 8 hpi that seemed to control virus growth effectively (Fig. 6B), since we could not detect any viral growth at later times postinfection. Consistent with this, the level of antiviral cytokines decreased progressively and reached a plateau at 16 hpi (Fig. 6B).

**FIG 7:**
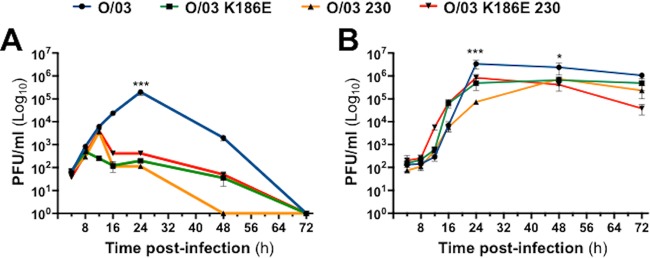
JAK1/2 inhibition restores growth kinetics of NS1 revertant viruses. E-derm cells were treated for 24 h with uIFN (A) or ruxolitinib (B) prior to infection (MOI, 0.1) with O/03, O/03-K186E, O/03-230, or O/03-K186E-230. Cells and supernatants were collected at different times postinfection. Viral titers were measured and significance was calculated as indicated in Materials and Methods. ***, *P* < 0.001, and *, *P* < 0.05 for O/03 at the indicated time postinfection versus the other three viruses at the same time postinfection. The error bars indicate SEM.

As the O/03 virus (but not the NS1 revertants) was able to replicate to high levels in the presence of antiviral cytokines, we wanted to determine if it was able to replicate in cells in an antiviral state induced by exogenous IFN. E-derm cells were pretreated with 500 U of uIFN for 24 h and then inoculated with O/03 and NS1 revertant viruses (MOI, 0.1). While all the viruses exhibited a reduction in virus growth compared to their observed replication abilities in untreated E-derm cells, the O/03 virus reached a titer of 10^5^ PFU/ml at 24 hpi, significantly higher than those of the other three viruses (Fig. 7A). These results show that the evolution of NS1 resulted in an increased ability to replicate in cells that had been exposed to uIFN.

To confirm the role of IFN in limiting the replication of the NS1 revertant viruses, we treated equine cells 24 h prior to infection (MOI, 0.1) with ruxolitinib (Selleck Chemicals) to block the JAK/STAT pathway and thus impaired the ability of the cells to respond to IFN (25). We compared the growth kinetics of each virus (Fig. 7B) in the presence of ruxolitinib. Although the O/03 virus maintained a significantly higher titer than the three other viruses at 24 and 48 hpi, the revertant viruses reached a higher titer than under normal conditions (Fig. 4B), confirming the central role of the IFN response in limiting the replication of O/03 NS1 revertant viruses in equine cells.

### The length of NS1 and the nature of residue 186 impact EIV-mediated control of ISG transcription and general gene expression.

**FIG 8:**
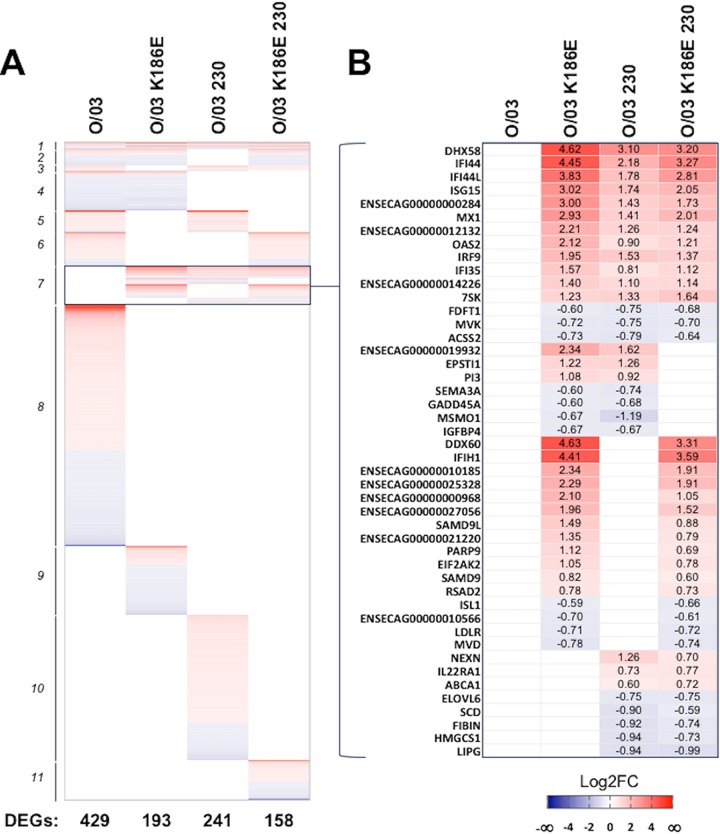
NS1 amino acid 186 and the C-terminal tail affect EIV control of gene expression in equine cells. (A) Heat map of DEGs between O/03- and revertant-virus-infected E-derm cells versus mock-infected samples. A ∣log_2_ FC∣ value of >0.58 and a *P* value of <0.05 were regarded as indicating statistically differential expression relative to mock infection. The total number of DEGs for each condition is indicated below the heat map. DEGs relative to mock-infected conditions were classified in 11 groups: 1, DEGs shared between O/03 and mutants; 2, DEGs shared between all viruses but O/03-230; 3, DEGs shared between all viruses but O/03-K186E; 4, DEGs shared between O/03 and O/03-K186E; 5, DEGs shared between O/03 and O/03-230; 6, DEGs shared between O/03 and O/03-K186E-230; 7, DEGs shared between all revertants; 8, DEGs present in O/03 only; 9, DEGs present in O/03-K186E only; 10, DEGs present in O/03-230 only; and 11, DEGs present in O/03-K186E-230 only. The complete list of genes in presented in Table S1 in the supplemental material. (B) DEGs (including some ISGs) present only in revertant virus-infected samples (group 7). The values are log_2_ FC. A ∣log_2_ FC∣ value of >0.58 and a *P* value of <0.05 were regarded as statistically significant.

To identify further effects of these adaptive mutations on virus-host interactions, we compared the transcriptomes of equine cells infected with the O/03 and revertant viruses. We first determined the total number of differentially expressed genes (DEGs) compared to mock-infected samples (Fig. 8) at 8 hpi (the end of the eclipse phase in E-derm cells) in order to avoid any bias due to different replication efficiencies between viruses. Cells infected with the O/03 virus exhibited the highest ratio of DEGs to mock-infected cells (*n* = 429), which correlates with increased production of proteins at 12 hpi (Fig. 5B) and likely reflects productive infection. Furthermore, the number of DEGs in revertant virus-infected cells compared to mock-infected samples decreased as follows: 241 for O/03-230-infected samples, 193 for O/03-K186E-infected samples, and 158 for O/03-K186E-230-infected samples (Fig. 8A; a detailed list of DEGs, including Gene ID, gene name, and log_2_-fold change, is shown in Table S1 in the supplemental material). This indicates that these two natural mutations in NS1 have a high impact on the response of equine cells to EIV infection, highlighting their roles in virus adaptation.

As the O/03 virus replicated to high titers despite inducing high levels of antiviral cytokines, we compared the number of ISGs and interferon-induced elements that were upregulated in cells infected with the revertant viruses but absent in O/03-infected samples (Fig. 8B). As expected, a large number of ISGs (i.e., ISG15, MX1, and OAS1/2/3) that were maintained at a basal level in cells infected with O/03 were upregulated in cells infected with the revertant viruses (Fig. 8B).

We also looked for variations in viral gene expression between O/03 and revertant viruses and did not observe any significant differences (data not shown).

### EIV NS1 evolved to block the induction of ISGs at a pretranscriptional level.

**FIG 9:**
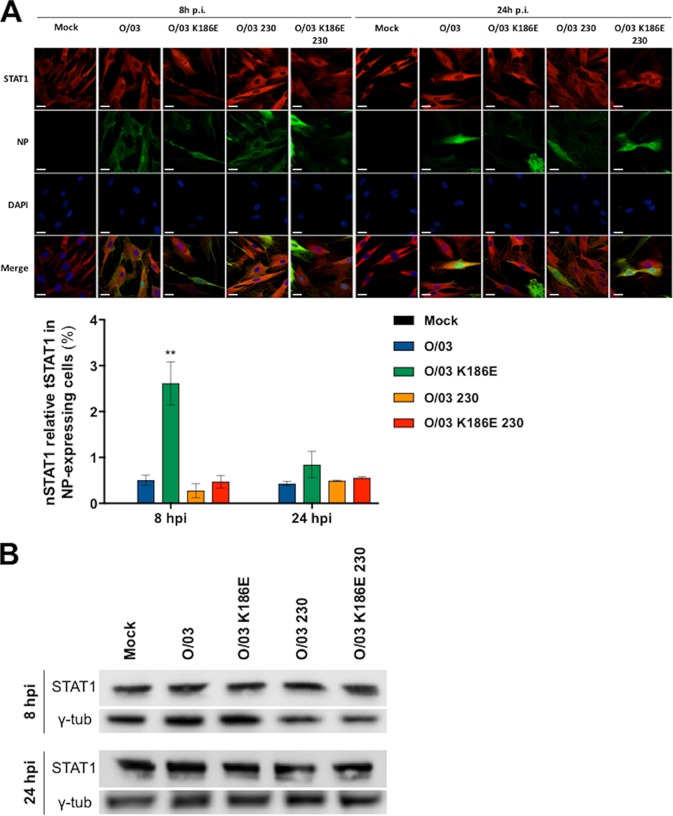
STAT1 nuclear localization in equine cells infected with EIV O/03 and NS1 revertant viruses. E-derm cells were infected (MOI, 0.1) with O/03, O/03-K186E, O/03-230, and O/03-K186E-230 viruses or mock infected for 8 or 24 h. (A) (Top) Confocal images showing coimmunostaining of viral NP and STAT1. The experiment was performed three times independently, and representative images of STAT1 and NP are shown. Scale bars, 20 μm. (Bottom) Graphical representation of nuclear localization of STAT1 (nSTAT1). nSTAT1 was quantified as described in Materials and Methods and expressed as a percentage of tSTAT1. Statistical significance was calculated as described in Materials and Methods. **, *P* < 0.01 for O/03-K186E versus other conditions at 8 hpi. No other significant differences were detected. The error bars indicate SEM. (B) Infected cells were lysed, and total STAT1 expression was determined by Western blotting. Gamma tubulin was used as a loading control.

Our reporter assays showed that the NS1 proteins that did not bind CPSF30 were able to control ISRE-containing promoters more efficiently (Fig. 1 to 3). The transcriptomics data showed that cells infected with O/03 (but not the revertants) exhibited significant control of ISG transcripts (Fig. 8B) despite inducing large amounts of antiviral cytokines (Fig. 6A). Furthermore, experiments using ruxolitinib treatment confirmed the central role of the JAK/STAT pathway in limiting revertant virus growth in equine cells (Fig. 7B). Taken together, these results suggested that NS1 of O/03 virus blocked the induction of ISGs at a pretranscriptional level and that this involved the JAK/STAT pathway, since the JAK/STAT signaling pathway, upon IFN stimulation, leads to the transcription of ISGs and this involves the nuclear translocation of the ISGF3 complex. As a marker of ISGF3 nuclear translocation, we compared the amounts of nuclear STAT1 at 8 and 24 hpi in equine cells infected with O/03 and revertant viruses. As expected, O/03 infection did not result in STAT1 nuclear localization, despite the presence of high levels of antiviral cytokines (Fig. 6A, 24 hpi). In addition, residue 186 played a central role in limiting STAT1 nuclear translocation, as O/03-K186E virus infection resulted in abundant nuclear STAT1 (Fig. 9A). STAT1 also remained in the cytoplasm of cells infected with O/03-230 or O/03-K186E-230 revertant virus, probably due to the low level of antiviral cytokines produced (Fig. 6C and D). However, we cannot rule out a possible contribution of the NS1 C-terminal tail to the control of STAT1 nuclear translocation. Furthermore, the observed differences were not due to different levels of expression of STAT1, as no significant differences in total STAT1 expression in cell lysates were detected by Western blotting (Fig. 9B). Finally, the percentages of infected E-derm cells at 8 hpi were comparable between O/03 and revertant viruses (data not shown), and a higher percentage of cells infected with O/03 was found at 24 hpi, further confirming the significant advantage of O/03 over all the revertant viruses after 24 hpi.

## DISCUSSION

We investigated the role of NS1 evolution in the posttransfer adaptation of an avian-origin IAV to a mammalian host, focusing on the roles of two mutations that appeared sequentially during EIV evolution. The E186K mutation appeared early after EIV emergence, while the 11-amino-acid C-terminal truncation appeared ∼20 years later. Our results showed that isogenic viruses carrying the evolved version of NS1 exhibit significantly higher fitness in equine cells. The O/03 virus from 2003 was able to replicate to high titers despite high levels of cytokines being produced by infected cells (Fig. 6A). This was achieved by blocking the induction of ISGs at a pretranscriptional level (Fig. 8B), which is consistent with the lack of STAT1 nuclear localization in infected cells (Fig. 9A). The ability to replicate in the presence of IFN is a significant fitness trait, as it not only renders an important arm of the host antiviral response ineffective, but also acts as a barrier to other respiratory viruses that might compete for the same ecological niche (i.e., the epithelium of the respiratory tract). Coinfection experiments will shed light on this issue.

In contrast, an isogenic virus carrying a revertant version of NS1 (O/03-K186E-230) that mimics the early, more avian-like NS1 proteins of EIV replicated to significantly lower titers over a 72-hour period in equine cells (Fig. 4B). This virus induced cellular-protein shutdown (Fig. 5B) and high levels of apoptosis (Fig. 5A), and this was associated with inefficient cell-to-cell spread (Fig. 4C). However, O/03-K186E-230 efficiently produced infectious particles at 12 hpi (Fig. 4B and 6D). This suggests that the emerging, nonadapted virus was highly susceptible to the host IFN response and therefore relied on blocking general gene expression nonspecifically to transiently control the innate immune response. However, this would likely affect cellular homeostasis and lead to premature apoptosis, depriving the virus of the cellular resources it needs to replicate. Thus, high replication efficiency during this early period (Fig. 4B) would enhance the chances of onward transmission.

**FIG 10:**
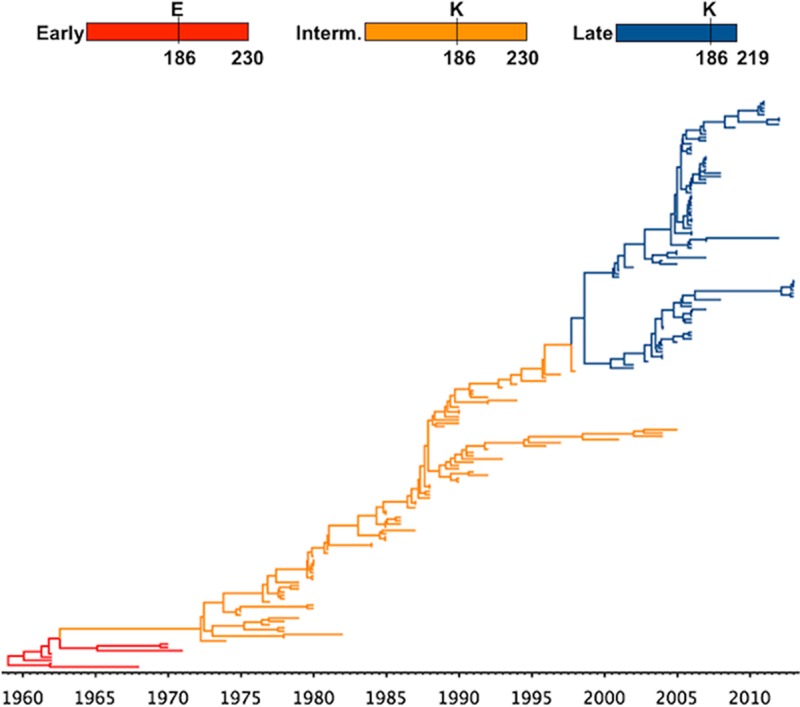
K186 and C-terminal truncation were fixed at the virus population level during EIV NS1 evolutionary history. Shown is a phylogenetic tree highlighting the fixation of E186K substitution and C-terminal truncation. The red branches represent NS1 proteins containing E186 and 230 amino acids (early NS1), the orange branches represent NS1 proteins containing K186 and 230 amino acids (intermediate NS1), and the blue branches represent NS1 proteins containing K186 and 219 amino acids (late NS1).

Early EIV NS1 proteins blocked general gene expression by binding to CPSF30, a host protein that plays a central role in pre-mRNA processing (12). A group of residues centered around residue 186 (184 to 188) (23, 26, 27), as well as other residues (103, 106, 108, and 125) (18, 19, 28), have been previously shown to be involved in this function. Interestingly, for EIV, that binding ability was lost with the introduction of K186, a substitution that must have been selectively advantageous for the virus. This is supported by the fact that it became quickly fixed at the virus population scale (Fig. 10) and because E186 in a contemporary EIV NS1 resulted in severe restriction of viral replication (Fig. 4B and 6B) and cell-to-cell spread (Fig. 4C). That single mutation impaired the ability of EIV to block the induction of the IFN response (Fig. 9A), resulting in the release of cellular cytokines (Fig. 6B) and subsequent upregulation of ISGs (Fig. 8B). These results are consistent with work we recently published on the evolution of the NS1 protein of H3N8 canine influenza virus, a virus that originated from the H3N8 EIV lineage in the early 2000s (29).

The second evolutionary change in NS1 that became rapidly fixed at the population level was a C-terminal truncation of the protein that appeared in the mid-1990s (Fig. 10). While determining the molecular mechanism underpinning the effect of the C-terminal tail was beyond the scope of this study, our experimental infections suggested that this truncation also increased viral fitness. Indeed, extending the tail of NS1 reduced virus replication (Fig. 4B and 6C) and plaque size (Fig. 4C), increased viral susceptibility to IFN (Fig. 7A and B), reduced viral control of ISG transcription (Fig. 8B) and expression of ISG15 and MX1 (Fig. 5A), and also resulted in early induction of apoptosis in infected cells (Fig. 5A). The last could be caused by the presence of a putative PDZ-binding domain in the tail of NS1 that has been previously associated with apoptosis (30, 31). Truncation of NS1 would therefore be beneficial for EIV, as it would provide better control of the IFN response and extend the life span of infected cells.

It is important to note that E186 is present in 98.2% of NS1 unique sequences derived from avian, human, swine, canine, and equine viruses (*n* = 12,775) (data not shown). However, K186 is more common among equine and canine IAVs. With regard to NS1 length, the majority (63.7%) of NS1 proteins are longer than 220 amino acids, and such “long” NS1 proteins are highly prevalent among IAVs derived from birds, humans, dogs, and horses, but not from pigs, as 77% of unique swine NS1 sequences are shorter than 220 residues. Further experimental work is required to unveil the roles of residue 186 and the C-terminal length of NS1 in the adaptation of IAVs to other species.

In summary, we have shown that mutations in the NS1 gene that became fixed during the continuous circulation of EIV in horses led to at least two temporally distinct changes in NS1 function that resulted in better control of the mammalian host innate immune response and likely contributed to the adaptation of an avian-origin virus to its new host, the horse. Comparisons of other mammalian IAV lineages of avian origin will show whether such dynamic strategies are common features of influenza A viruses while adapting to mammals.

## MATERIALS AND METHODS

### Cells.

MDCK (ATCC CCL-34) and human embryonic kidney (293T; ATCC CRL-11268) cells were grown at 37°C and 5% CO_2_ in Dulbecco's modified Eagle's medium (DMEM) high glucose, GlutaMax, and pyruvate (ThermoFisher Scientific) supplemented with 10% fetal calf serum (Gibco Life Technologies) and 1% PS (100 units/ml penicillin, 100 μg/ml streptomycin; Gibco Life Technologies). E-derm cells (ATCC CCL-57) were grown at 37°C and 5% CO_2_ in DMEM high glucose, GlutaMax, and pyruvate supplemented with 15% fetal calf serum (Gibco Life Technologies), 1% nonessential amino acids (Gibco, Life Technologies), 1% PS (Gibco Life Technologies).

### Viruses.

Viral stocks (accession number; abbreviation) of A/equine/Uruguay/1/1963 (ACD85423; U/63), A/equine/Miami/1/1963 (ABY81497; M/63), A/equine/SaoPaulo/1/1969 (ACD85390; SP/69), A/equine/Fontainebleau/1/1979 (ACD85401; F/79), A/equine/Sussex/1/1989 (ACD97430; S/89), A/equine/Kentucky/1/1991 (ACA24672; K/91), A/equine/LaPlata/1995 (MF182460; LP/95), A/equine/Kentucky/1995 (MF182451; K/95), A/equine/Kentucky/1999 (MF182443; K/99), A/equine/Kentucky/5/2002 (ABA42429; K/02), A/equine/Newmarket/5/2003 (ACI48802; N/03), A/equine/Ohio/1/2003 (ABA42431; O/03), and A/equine/Mongolia/3/2013 (MF182459; M/13) were grown at passage 2 in MDCK cells (MOI, 0.1) and then aliquoted and stored at −80°C.

Reverse genetic influenza A/equine/Ohio/1/03 (O/03), H3N8 O/03, and NS1 (O/03-K186E, O/03-230, and O/03-K186E-230) revertant viruses were grown in MDCK cells at 37°C and 5% CO_2_. For infections, virus stocks were diluted in infection medium (DMEM, 0.3% bovine serum albumin [BSA], 1% PS, and 1 μg/ml of tosylsulfonyl phenylalanyl chloromethyl ketone [TPCK]-treated trypsin [Sigma]) (32).

SeV strain Cantell was purchased from Charles River Laboratories and stored at −80°C. The rVSV-GFP virus stock used in the bioassay was generated by transiently transfecting HEK293T cells in Opti-MEM with an expression plasmid encoding the VSV surface glycoprotein (pVSV-G) (24) using TransIT-LT1 transfection reagent (Cambridge Biosciences) at 37°C and 5% CO_2_ for 36 h (100-mm sterile-dish format; 5 × 10^6^ cells). At the end of the transfection period, the cells were infected with a previous stock of rVSV-GFP (2.5 × 10^2^ 50% tissue culture infective doses [TCID_50_]) for 4 h at 37°C and 5% CO_2_ in growth medium and then washed with phosphate-buffered saline (PBS) and maintained at 37°C and 5% CO_2_ in growth medium for a further 16 h. The supernatant was then collected and filtered through a 0.45-μm filter (Fisher Scientific) before being aliquoted and stored at −80°C.

### Cloning of NS1.

Mammalian expression constructs for untagged NS1 used in the reporter assay were generated as previously described (18, 19). pcDNA3 plasmids encoding NS1 were also generated by subcloning from pCAGGS (18). The sequence of each pCAGGS- or pcDNA3-NS1 insert was confirmed by Sanger sequencing and compared to those available in the NCBI Influenza Database. Viral RNA (vRNA) was extracted from the corresponding viral stocks using the QIAmp viral RNA kit (Qiagen), the RNA quality was assessed with a NanoDrop 2000 spectrophotometer (ThermoFisher Scientific), and the RNA was stored at −80°C. Reverse transcription (RT) was done using Uni12 primer (33), 500 ng of RNA, and SuperScript III reverse transcriptase (Invitrogen) following the manufacturer's protocol. PCR was done with 100 ng of cDNA and PfuUltra II fusion HS DNA polymerase (Agilent), following the manufacturer's protocol, and a specific set of primers was used for each NS1. The cycling parameters and primers are available upon request. In order to prevent expression of NEP in the pCAGGS-NS1 plasmids, a silent mutation in the splice acceptor site of NS1 (splice acceptor mutation [SAM]) was introduced for each construct as previously described (18, 19).

To engineer the three NS1 revertants, lysine (K)-to-glutamic acid (E) (codon 186, AAA changed to GAA) and stop codon-to-arginine (R) (codon 220, TGA replaced by CGA) mutations were introduced into the pCAGGS-splice acceptor revertant O/03 NS1 constructs and the ambisense pDP2002 plasmid carrying the O/03 NS gene using PFUTurbo DNA polymerase (Agilent) and specific sets of primers. The presence of introduced mutations was confirmed by Sanger sequencing. The cycling parameters and primers are available upon request.

### Reporter assay.

For analysis of IFN-β and ISRE promoter activation and general gene expression, 293T cells (12-well plate format; 2.5 × 10^5^ cells/well) were transiently cotransfected using TransIT-LT1 transfection reagent (Cambridge Biosciences), with either 50 ng of pIFN-β-FF-Luc or 50 ng of pISRE-FF-Luc (reporter plasmids encoding FF-Luc under the control of the IFN-β promoter or the ISRE promoter, respectively) and 50 ng of a plasmid constitutively expressing Renilla luciferase (pREN-Luc) under the simian virus 40 (SV40) promoter (24) (kindly provided by Benjamin G. Hale), as well as 1,000 ng of the indicated pCAGGS-SAM NS1-expressing plasmids (or empty pCAGGS; 1,000 ng). After 24 h of transfection, the cells were infected with 50 hemagglutinating units (HAU) of SeV for 18 h and then lysed with 250 μl of passive lysis buffer (Promega). IFN-β–FF-Luc, ISRE-FF-Luc, and REN-Luc activities were measured using the dual-luciferase reporter assay system (Promega), as directed by the manufacturer's protocol. All transfections were carried out in triplicate, and experiments were repeated independently three times.

### Coimmunoprecipitation of NS1 with CPSF30.

Equine CPSF30 was cloned as previously described for human CPSF30 (18). Briefly, the equine CPSF30 gene was amplified by RT-PCR from equine cells using oligo(dT) and PCR with specific primers. The RT-PCR product was then cloned into the pCAGGS HA-COOH plasmid as described previously (34). NS1 proteins were synthesized *in vitro* using pcDNA3 plasmids and the TNT7 transcription/translation kit (Promega), following the manufacturer's recommendations. Human 293T cells (1.5 × 10^6^ cells/well; 6-well format; triplicates) were transiently transfected with 2 μg/well of an HA-equine CPSF30-expressing pCAGGS plasmid. Forty-eight hours posttransfection, the cells were lysed in 20 mM Tris-HCl (pH 7.5), 100 mM NaCl, 0.5 mM EDTA, 5% glycerol, 1% Triton X-100 supplemented with a complete mini-protease inhibitor cocktail (Pierce). Cleared cell lysates were incubated overnight at 4°C with the *in vitro*-synthesized O/03 and revertant NS1 proteins and 20 μl of an anti-HA affinity resin (Sigma). After extensive washing, precipitated proteins were dissociated from the resin using Laemmli buffer plus β-mercaptoethanol. Total proteins from cell lysates were then analyzed by SDS-PAGE and Western blotting as described below, using a primary specific rabbit polyclonal antibody against NS1 (GenScript) or an HA tag for CPSF30 (Sigma).

### Rescue of recombinant H3N8 EIVs.

Viruses were rescued as previously described (35). Briefly, cocultures (2:3) of 293T and MDCK cells (6-well plate format; 2 × 10^6^ cells/well) were seeded 24 h prior to viral rescue. The cells were transiently cotransfected using TransIT-LT1 (Cambridge Bioscience) with 2.5 μg of seven ambisense O/03 plasmids (pDP2002-PB2, -PB1, -PA, -HA, -NP, -NA, and -M) plus ambisense O/03 NS plasmid (O/03) or the NS revertant constructs (O/03-K186E, O/03-230, and O/03-K186E-230). At 24 hpt, the medium was replaced by infection medium. Virus-containing tissue culture supernatants were collected 2 or 3 days posttransfection, clarified, and used to infect fresh MDCK cells (P1 stock) for 2 to 3 days postinfection. Viral titers of P1 stocks were determined by immunofocus assay (focus forming units [FFU] per milliliter) in MDCK cells, using mouse monoclonal anti-NP antibody (clone HB-65; European Veterinary Laboratory), horseradish peroxidase (HRP)-conjugated rabbit anti-mouse IgG antibody (AbD Serotec), and TrueBlue peroxidase substrate (Insight Biotechnology), as previously described (35). These P1 stocks were then used to grow P2 viral stocks at an MOI of 0.01 in MDCK cells for further use in experiments. For experimental infections, a minimum of two viral stocks for each virus were rescued and grown independently. The NS segment of each virus was sequenced by the Sanger method to confirm the sequence of the O/03 or revertant viruses.

### Viral infection.

Confluent monolayers of MDCK cells (12-well plate format; triplicates; 5 × 10^5^ cells/well) or E-derm cells (12-well plate format; triplicates; 2.5 × 10^5^ cells/well) were infected (MOI, 0.01 and 0.1, respectively) with the indicated viruses and placed at 37°C and 5% CO_2_. The cells were grown on coverslips for confocal microscopy. After 1 h of incubation, the cells were washed with PBS, and the infection medium was replaced with 500 μl of fresh growth medium. Tissue culture supernatants were collected at various times postinfection and stored at −80°C, and the cells were fixed in 0.1% buffered formalin at 4°C for 16 h and kept for confocal microscopy or fluorescence-activated cell sorting (FACS) analysis. Each experiment was repeated three times independently. Viral titers were determined by immunofocus assay in MDCK cells. Titrations were repeated three times independently, and the mean value and standard error of the mean (SEM) were calculated using GraphPad Prism 7.

To measure virus growth kinetics in the presence of an inhibitor of the IFN response, E-derm cells were treated with ruxolitinib. Ruxolitinib was prepared as 10 mM stocks in dimethyl sulfoxide (DMSO) and used at a concentration of 4 μM (25). Treatment was started 24 h prior to infection and maintained at the same concentration for the whole experiment.

For plaque phenotype, a confluent monolayer of MDCK cells (6-well plate format; triplicates; 6.4 × 10^4^ cells/well) were infected with serial dilutions (1:2) of the viral stock of interest and placed for incubation at 37°C and 5% CO_2_ in a humidified atmosphere. The plates were gently rocked every 10 min. After 1 h of incubation, the cells were gently washed with PBS, and the infection medium was replaced with a 50:50 2.4% Avicell-2× minimal essential medium (MEM) overlay for exactly 48 h. At 48 hpi, the overlay was discarded, and the cells were gently washed 3 times with PBS and fixed with 80% ice-cold acetone solution (Sigma-Aldrich) for 10 min at room temperature. The plates were then allowed to dry overnight at room temperature before being treated with 1% Triton X-100 (Sigma-Aldrich)-PBS solution for 10 min at room temperature, followed by 1 h of incubation with 10% normal goat serum (NGS)-PBS solution at room temperature. This was followed by overnight immunoblotting at 4°C in 10% normal goat serum plus PBS with a monoclonal anti-influenza A virus NP antibody (clone HB65; European Veterinary Laboratory; 1/500 dilution) under gentle agitation. After a 3-step washing with PBS, a horseradish peroxidase-conjugated rabbit anti-mouse IgG antibody (AbD Serotec, United Kingdom; 1/1,000 dilution) was used in PBS solution for a further 4 h at room temperature under gentle agitation. A color development method was used to reveal the immunofocus using the TrueBlue peroxidase substrate (Insight Biotechnology); 2 ml of substrate was used per well for exactly 10 min before being stopped with tap water.

### Viral and cellular protein staining for FACS and confocal microscopy.

Cells were permeabilized with 1% Triton X-100 for 10 min and blocked in PBS-10% normal goat serum (Gibco, Life Technologies) for 1 h. The cells were then incubated with anti-influenza A virus NP antibody, rabbit polyclonal anti-NS1 protein antibody (GenScript), or rabbit STAT1 polyclonal antibody (Santa Cruz) overnight at 4°C. The cells were then washed twice with PBS and incubated for 4 h with rabbit anti-mouse IgG Alexa Fluor 488 (Cell Signaling) or donkey anti-rabbit IgG Alexa Fluor 555 (Cell Signaling) before FACS analysis (Guava flow cytometer; Merck) or fixed in Vectashield antifade mounting medium with DAPI (4′,6-diamidino-2-phenylindole) (Vector Laboratories) and analyzed by confocal microscopy.

For confocal microscopy, images were taken with a 63× oil objective on a Zeiss LSM 880 confocal microscope with gallium arsenide phosphide (GaAsP) detector. All images were taken with cross talk minimized using best signal calculations in Zen software; 3 by 3 tilescans were collected in three different positions for each sample. The experiment was repeated three times independently. The images were imported into ImageJ, and STAT1 nuclear localization was analyzed in NP-positive cells, with a minimum of 1,000 NP-expressing cells analyzed for each sample. The nuclear localization of STAT1 was analyzed using the AND function in the image calculator and was accomplished by detecting colocalization signals between STAT1 and DAPI, using the colocalization threshold function in ImageJ. The quantity of nuclear STAT1 (nSTAT1) was expressed as a percentage of total STAT1 (tSTAT1) in infected cells. For the representative pictures of nSTAT1, the signal intensity of nSTAT1 was normalized to O/03-K186E at 8 hpi.

### SDS-PAGE and Western blotting.

Cells were lysed in Laemmli buffer plus β-mercaptoethanol and stored immediately at −80°C. Samples were boiled for 15 min at 95°C prior to polypeptide separation by SDS-PAGE on NuPAGE Novex 4 to 12% Bis-Tris protein gels (ThermoFisher Scientific). Proteins were detected by Western blotting following transfer to nitrocellulose membranes. The membranes were blocked for 1 h at room temperature in 5% milk–Tris-buffered saline with 0.1% Tween 20 (TBST) and immunoblotted overnight at 4°C in 5% milk-TBST (0.1% Tween 20) with the following antibodies: mouse monoclonal anti-Mx1 (clone M143; provided by Georg Kochs, University of Freiburg, Freiburg, Germany), rabbit polyclonal anti-ISG15 (Proteintech), rabbit polyclonal anti-NS1 (GenScript), rabbit monoclonal anti-cleaved caspase-3 and rabbit monoclonal anti-caspase-3 (Cell Signaling), rabbit monoclonal anti-γ-tubulin (Sigma), mouse monoclonal anti-puromycin (clone 12D10; Millipore), HRP-conjugated anti-mouse IgG (Dako), and HRP-conjugated anti-rabbit IgG (Dako). The chemiluminescent signal was detected using Amersham ECL Prime Western blotting detection reagent (GE Healthcare) and captured with a ChemiDoc XRS+ system (Bio-Rad).

### Antiviral cytokine production and general protein shutdown.

E-derm cells (12-well plate format; triplicates; 2.5 × 10^5^ cells/well) were infected (MOI, 0.1) with the indicated viruses for a total of 72 h. At the indicated times postinfection, the supernatants were collected and stored at −80°C for further analysis by bioassay, while the cells were treated for 1 h at 37°C and 5% CO_2_ with puromycin (20 μg/ml in DMEM, 15% FBS) prior to lysis in Laemmli buffer plus β-mercaptoethanol and stored at −80°C for further analysis by Western blotting. Puromycin is a well-known antibiotic that competes against aminoacyl tRNA on the ribosome A site (36). As such, puromycin enables examination of total protein production without requiring transfection, radiolabeling, or the prior choice of a candidate gene (37). If cells are incubated with puromycin, lysed, and immunoblotted using an anti-puromycin antibody, all the proteins produced will be immunostained, as puromycin is incorporated at the C termini of all the nascent proteins. For the bioassay, supernatants were UV inactivated for 5 min at room temperature and used to treat fresh E-derm cells (48-well plate format; 6 × 10^4^ cells/well; triplicates) for 24 h. The cells were then infected with rVSV-GFP (2.5 × 10^2^ TCID_50_) for 8 h and then trypsinized and fixed in 0.1% buffered formalin for 16 h at 4°C. The percentage of GFP-expressing cells was then analyzed by FACS. For controls, E-derm cells were mock treated or treated with 500 units of uIFN. The GFP expression of mock-treated cells infected with rVSV-GFP was considered 100%, and the GFP expression of uIFN-treated cells infected with rVSV-GFP was considered 0%. Mean values and standard errors of the mean were calculated with GraphPad Prism 7.

### Transcriptome sequencing (RNA-seq).

Confluent monolayers of E-derm cells (12-well plates; triplicates; 2.5 × 10^5^ cells/well) were infected with O/03 and revertant viruses (MOI, 1) or mock infected at least three times independently. At 8 hpi, the cells were washed with PBS and immunostained with anti-NP antibody, and the proportion of infected cells was determined by FACS. The samples containing similar proportions of infected cells were selected for transcriptomic analysis, and the cells were lysed with 500 μl of TRIzol (ThermoFisher Scientific) for further RNA extraction.

Total RNA was extracted using the TRIzol method and further purified using RNeasy mini-spin columns (Qiagen), including an on-column DNase I digestion step (Qiagen) according to the manufacturer's protocol. The RNA concentration was measured with Qubit and the Qubit RNA HS assay kit (ThermoFisher Scientific) following the manufacturer's protocol. The rRNA integrity number was measured using an Agilent 2100 BioAnalyzer (Agilent Technologies).

Total RNA (4.5 μg) was enriched by selectively depleting rRNA using the RiboMinus Eukaryote kit v2 (Ambion, Life Technologies). The sequence reads (BioProject accession number PRJEB21264) were processed according to the Tuxedo pipeline (38). Read quality was assessed using FastQC, and TopHat2 and Bowtie2 were used to map short reads against the Equus caballus 2 genome (GCA_000002305.1). A list of differentially expressed genes compared to mock-infected samples was generated using CuffDiff2 (genes with Benjamini Hochberg *P* values of <0.05 were considered significant) (39).

### Phylogenetic analysis of EIV NS1 sequences.

We collected 170 EIV H3N8 NS sequences from the NCBI Influenza Virus Resource database (the sequences are available upon request). We also included four EIV H3N8 NS sequences that we sequenced for this study (accession numbers MF182460, MF182451, MF182443, and MF182459) (40). SeaView version 4.6.1 (41) was used to align the NS1 coding regions, and the final alignment was edited manually. NEP coding regions were removed from the alignment. We used BEAST version 1.8.4 (42) to infer maximum clade credibility (MCC) trees. We used a strict molecular clock and an HKY85+G model of nucleotide substitution. Each codon position was estimated with separate substitution rates and nucleotide frequencies. Two individual chains were run until convergence was achieved.

### Statistical analysis.

Unless otherwise indicated, significance was calculated by two-way analysis of variance (ANOVA), followed by Bonferroni's multiple-comparison test.

### Accession number(s).

Data determined in this study may be found in the NCBI BioProject database under accession number PRJEB21264 and in GenBank under accession numbers MF182460, MF182451, MF182443, and MF182459.

## Supplementary Material

Supplemental material
