# Supplementary material for: Mammalian Adaptation of an Avian Influenza A Virus Involves Stepwise Changes in NS1
Source: J Virol. 2018 Feb 12;92(5):e01875-17. doi: 10.1128/JVI.01875-17 (PMC5809720; doi:10.1128/JVI.01875-17)
Supplement: Supplemental material [file supp_92_5_e01875-17__index.html]

Supplemental material 

# Mammalian Adaptation of an Avian Influenza A Virus Involves Stepwise Changes in NS1

## Supplemental material

- Supplemental file 1 -

  Table S1 (Differentially expressed genes in E-derm cells infected with the indicated viruses.)

  PDF, 254K
