# Supplementary material for: Mammalian Adaptation of an Avian Influenza A Virus Involves Stepwise Changes in NS1
Source: J Virol. 2018 Feb 12;92(5):e01875-17. doi: 10.1128/JVI.01875-17 (PMC5809720; doi:10.1128/JVI.01875-17)
Supplement: Supplemental material [file JVI.01875-17_zjv005183342s1.pdf]

Table S1. List of differentially expressed genes (including Gene ID, gene name, and log2 fold change) in E-derm cells infected with the indicated viruses.

		Viruses	O/03		O/03-K186E		O/03-230		O/03-K186E-230	
DEG groups	Gene ID	Gene name	Rank	Log2FC	Rank	Log2FC	Rank	Log2FC	Rank	Log2FC
Group 1	ENSECAG00000010153	IFIT3	Up15	2.8169	Up2	5.21902	Up3	3.73116	Up3	4.28147
	ENSECAG00000027377	5_8S_rRNA	Up39	1.31487	Up12	2.86602	Up14	1.38835	Up15	1.73267
	ENSECAG00000023135	LYRM7	Up53	1.06639	Up57	0.740132	Up43	0.941237	Up34	1.03292
	ENSECAG00000004574	ENSECAG00000004574	Up41	1.23114	Up37	1.14136	Up53	0.879453	Up37	0.941438
	ENSECAG00000004433	IFIT1	Up104	0.771454	Up7	4.13469	Up5	2.51653	Up9	3.1852
	ENSECAG00000008899	LSS	Down137	-0.624569	Down31	-0.820577	Down11	-0.962401	Down5	-1.00929
	ENSECAG00000009202	TYSND1	Down27	-0.907853	Down18	-0.942333	Down32	-0.740013	Down48	-0.615778
	ENSECAG00000004572	CH25H	Down12	-1.13537	Down10	-1.04437	Down9	-1.01427	Down8	-0.977711
	ENSECAG00000008688	ENSECAG00000008688	Down100	-0.673453	Down101	-0.611868	Down2	-1.23933		
Group 2	ENSECAG00000025867	U3	Up17	2.40357	Up15	2.44815			Up5	3.37152
	ENSECAG00000019095	ENSECAG00000019095	Up46	1.16439	Up49	0.866737			Up56	0.704857
	ENSECAG00000027676	ENSECAG00000027676	Up57	1.02918	Up35	1.1478			Up27	1.16402
	ENSECAG00000016730	ENSECAG00000016730	Up85	0.84377	Up43	0.966973			Up39	0.893828
	ENSECAG00000003015	SERPINB2	Up151	0.676015	Up58	0.684345			Up83	0.61405
	ENSECAG00000019165	CENPK	Up177	0.649676	Up65	0.581247			Up45	0.823367
	ENSECAG00000016459	ENSECAG00000016459	Up190	0.642261	Up64	0.594166			Up89	0.609109
	ENSECAG00000004107	DDIT4	Down175	-0.586586	Down103	-0.608796			Down25	-0.716045
	ENSECAG00000000429	POLRMT	Down157	-0.604098	Down82	-0.650021			Down47	-0.61954
	ENSECAG00000019125	FASN	Down155	-0.605124	Down32	-0.819688			Down27	-0.713131
	ENSECAG00000012635	BRAT1	Down146	-0.611826	Down86	-0.641948			Down29	-0.692893
	ENSECAG00000004005	MESDC1	Down134	-0.627071	Down37	-0.785754			Down49	-0.608919
	ENSECAG00000018860	ZFP36L1	Down113	-0.653149	Down84	-0.64632			Down51	-0.60197
	ENSECAG00000001989	SESN2	Down88	-0.698381	Down94	-0.62295			Down32	-0.682113
	ENSECAG00000000607	TRIB3	Down50	-0.800805	Down38	-0.781903			Down11	-0.833385
	ENSECAG00000013176	MEGF6	Down39	-0.842357	Down80	-0.665181			Down37	-0.664524
	ENSECAG00000009989	CHAC1	Down29	-0.901175	Down27	-0.849656			Down23	-0.72979
	ENSECAG00000011234	COL7A1	Down26	-0.926848	Down89	-0.636524			Down33	-0.675143
	ENSECAG00000011475	ENSECAG00000011475	Down17	-1.05965	Down9	-1.08052			Down9	-0.924415
Group 3	ENSECAG00000009190	TMSB4X	Up58	1.02308			Up15	1.3809	Up68	0.658129
	ENSECAG00000021345	POLR2K	Up51	1.08059			Up22	1.25657	Up72	0.646725
	ENSECAG00000009383	TBCA	Up77	0.919505			Up13	1.39272	Up79	0.62422
	ENSECAG00000013756	C8orf59	Up82	0.869592			Up87	0.730823	Up50	0.734264
	ENSECAG00000020689	SPA17	Up197	0.633619			Up135	0.642672	Up94	0.600385
	ENSECAG00000014624	SPX	Up243	0.586334			Up119	0.671073	Up87	0.61023
Group 4	ENSECAG00000020860	LSMEM1	Up13	3.19098	Up14	2.51879				
	ENSECAG00000022299	LRRC66	Up28	1.59154	Up33	1.20033				
	ENSECAG00000004180	HSPA6	Up31	1.50962	Down5	-1.31121				
	ENSECAG00000026824	LYRM4	Up96	0.817396	Up53	0.764381				
	ENSECAG00000026993	CEBPZOS	Up116	0.749826	Up56	0.740731				
	ENSECAG00000013685	TNXB	Down170	-0.588493	Down115	-0.594876				
	ENSECAG00000006963	NACC2	Down156	-0.604823	Down97	-0.620019				
	ENSECAG00000021411	TM7SF2	Down147	-0.611736	Down47	-0.73979				
	ENSECAG00000024890	CCDC120	Down141	-0.616803	Down56	-0.72215				
	ENSECAG00000012684	SWSAP1	Down138	-0.623099	Down98	-0.615043				
	ENSECAG00000010395	RIN1	Down130	-0.636608	Down99	-0.614772				
	ENSECAG00000010506	PLEKHH3	Down124	-0.643436	Down125	-0.58471				
	ENSECAG00000018552	CDKN2A	Down120	-0.646724	Down95	-0.621609				
	ENSECAG00000001181	CBX4	Down119	-0.647958	Down119	-0.591799				
	ENSECAG00000006501	FJX1	Down118	-0.649401	Down121	-0.590583				
	ENSECAG00000013443	AKNA	Down117	-0.649643	Down118	-0.592903				
	ENSECAG00000016720	OBSCN	Down111	-0.654409	Down120	-0.59137				
	ENSECAG00000012169	ATP5D	Down110	-0.656075	Down93	-0.623178				
	ENSECAG00000020898	RELB	Down104	-0.663394	Down104	-0.606461				
	ENSECAG00000011704	TNFAIP2	Down103	-0.667399	Down29	-0.834622				
	ENSECAG00000015306	ATG4D	Down97	-0.682022	Down81	-0.658705				
	ENSECAG00000006302	SLC25A29	Down93	-0.688544	Down53	-0.729061				
	ENSECAG00000013820	KMT5C	Down86	-0.702321	Down39	-0.779386				
	ENSECAG00000021748	ENSECAG00000021748	Down80	-0.705971	Down23	-0.864304				
	ENSECAG00000003315	IRS2	Down79	-0.707088	Down85	-0.64472				
	ENSECAG00000021641	SNX21	Down75	-0.718118	Down46	-0.744673				
	ENSECAG00000016064	SYDE1	Down74	-0.720635	Down54	-0.725743				
	ENSECAG00000017274	KRT13	Down72	-0.722815	Down100	-0.613584				
	ENSECAG00000019910	PDLIM2	Down69	-0.728577	Down61	-0.710742				
	ENSECAG00000023023	OSGIN1	Down57	-0.762054	Down43	-0.765127				
	ENSECAG00000013201	MMP17	Down51	-0.794045	Down30	-0.821235				
	ENSECAG00000013281	IRGQ	Down47	-0.804184	Down19	-0.928778				
	ENSECAG00000014596	GYLTL1B	Down44	-0.815025	Down15	-0.974631				
	ENSECAG00000003075	BORCS6	Down43	-0.818891	Down11	-1.03005				
	ENSECAG00000019726	ENSECAG00000019726	Down42	-0.819503	Down12	-1.01958				
	ENSECAG00000003591	TPGS1	Down38	-0.84294	Down67	-0.694915				
	ENSECAG00000020219	ENSECAG00000020219	Down36	-0.852069	Down20	-0.920106				

	ENSECAG00000006082	ENSECAG00000006082	Down34	-0.863856	Down22	-0.866239				
	ENSECAG00000010241	SPSB4	Down33	-0.867167	Down70	-0.691728				
	ENSECAG00000021543	CDKN1A	Down25	-0.933939	Down17	-0.954175				
	ENSECAG00000004462	HIC1	Down21	-0.989624	Down63	-0.701717				
	ENSECAG00000005956	TPBGL	Down20	-1.00602	Down13	-1.01796				
	ENSECAG00000019043	SSC5D	Down15	-1.07302	Down21	-0.888949				
	ENSECAG00000000154	SDHAF1	Down14	-1.07843	Down3	-1.38192				
	ENSECAG00000011185	BTBD19	Down8	-1.22584	Down14	-1.00361				
	ENSECAG00000006098	ANKRD9	Down2	-1.95687	Down1	-1.94064				
Group 5	ENSECAG00000010664	GNRH1	Up2	inf			Up1	inf		
	ENSECAG00000025973	U1	Up8	inf			Up2	inf		
	ENSECAG00000016983	ANKRD1	Up47	1.14911			Up29	1.1145		
	ENSECAG00000011446	ENSECAG00000011446	Up72	0.932911			Up19	1.26822		
	ENSECAG00000025120	SBSN	Up86	0.843737			Up34	1.06734		
	ENSECAG00000018861	ENSECAG00000018861	Up91	0.827969			Up100	0.713307		
	ENSECAG00000020428	ENSECAG00000020428	Up110	0.760672			Up37	0.989556		
	ENSECAG00000013843	TUBE1	Up114	0.752447			Up62	0.828848		
	ENSECAG00000021551	UTP11L	Up119	0.74427			Up88	0.730403		
	ENSECAG00000018544	SPDL1	Up121	0.740986			Up164	0.599705		
	ENSECAG00000000651	SPINK5	Up133	0.709617			Up47	0.910982		
	ENSECAG00000000708	SARNP	Up134	0.709311			Up150	0.627748		
	ENSECAG00000002607	WBP5	Up144	0.695514			Up27	1.14543		
	ENSECAG00000010777	FAM107B	Up154	0.672125			Up38	0.989344		
	ENSECAG00000007912	HMGN4	Up156	0.670885			Up56	0.869776		
	ENSECAG00000015007	SKA2	Up162	0.665233			Up90	0.729377		
	ENSECAG00000020945	MNS1	Up193	0.639925			Up33	1.07119		
	ENSECAG00000019024	FAM204A	Up196	0.636851			Up157	0.604718		
	ENSECAG00000003783	ENSECAG00000003783	Up199	0.630491			Up138	0.639737		
	ENSECAG00000026937	SNRNP48	Up202	0.626273			Up147	0.629964		
	ENSECAG00000006773	CIR1	Up221	0.609636			Up45	0.914679		
	ENSECAG00000024519	SPC25	Up232	0.598761			Up179	0.585074		
	ENSECAG00000026920	HSBP1	Up236	0.59354			Up111	0.6827		
	ENSECAG00000011948	FIGF	Down81	-0.704733			Down19	-0.841851		
	ENSECAG00000024260	TMEM160	Down52	-0.793587			Down13	-0.937601		
	ENSECAG00000001022	UCN2	Down13	-1.08918			Up61	0.835742		
Group 6	ENSECAG00000019576	ENSECAG00000019576	Up3	inf					Up1	inf
	ENSECAG00000020819	AGER	Up26	1.65782					Up18	1.53619
	ENSECAG00000005401	ENSECAG00000005401	Up32	1.48633					Up22	1.29573
	ENSECAG00000012348	EFNB2	Up33	1.45556					Up30	1.08431
	ENSECAG00000014338	EGR1	Up35	1.38557					Up23	1.27712
	ENSECAG00000015342	CXCL8	Up40	1.30399					Up31	1.08159
	ENSECAG00000007744	RSRP1	Up44	1.1724					Up36	0.979854
	ENSECAG00000017181	PTGS2	Up67	0.954572					Up41	0.868289
	ENSECAG00000001334	C3orf14	Up75	0.922307					Up64	0.671459
	ENSECAG00000005903	ENSECAG00000005903	Up80	0.899233					Up44	0.825845
	ENSECAG00000003755	PIGW	Up87	0.833383					Up92	0.600669
	ENSECAG00000007070	TPRKB	Up101	0.80597					Up69	0.657097
	ENSECAG00000025162	ATP5EP2	Up115	0.752403					Up78	0.631377
	ENSECAG00000024911	ITGB3BP	Up118	0.746261					Up82	0.61876
	ENSECAG00000010468	CHCHD7	Up120	0.743429					Up98	0.597031
	ENSECAG00000027699	ENSECAG00000027699	Up129	0.714441					Up42	0.82849
	ENSECAG00000018495	SSB	Up130	0.71181					Up93	0.60062
	ENSECAG00000019053	FAM175A	Up136	0.7075					Up62	0.681148
	ENSECAG00000026862	GCSH	Up145	0.69445					Up91	0.606816
	ENSECAG00000011843	ENSECAG00000011843	Up153	0.673509					Up66	0.664985
	ENSECAG00000018894	RPL22L1	Up161	0.665869					Up67	0.664411
	ENSECAG00000027675	ND4	Up170	0.653594					Up52	0.726651
	ENSECAG00000011465	ISCA1	Up184	0.645685					Up84	0.613693
	ENSECAG00000020965	CMC1	Up191	0.641895					Up99	0.594351
	ENSECAG00000027684	ND1	Up200	0.626917					Up49	0.735849
	ENSECAG00000020387	MRPS18C	Up204	0.625188					Up59	0.6987
	ENSECAG00000016939	RPS24	Up212	0.619026					Up81	0.621074
	ENSECAG00000017538	LY96	Up215	0.614693					Up75	0.634798
	ENSECAG00000020416	ARPC3	Up225	0.604788					Up96	0.598332
	ENSECAG00000020144	UBXN2A	Up237	0.590855					Up100	0.594333
	ENSECAG00000023484	SKA1	Up244	0.585847					Up76	0.633546
	ENSECAG00000009792	UNC5B	Down160	-0.598728					Down22	-0.734704
	ENSECAG00000023637	ADAM33	Down135	-0.626909					Down36	-0.666905
	ENSECAG00000007338	SCARF2	Down106	-0.66066					Down54	-0.593362
	ENSECAG00000018651	GTPBP3	Down96	-0.682899					Down30	-0.684212
	ENSECAG00000003436	APBB3	Down65	-0.736995					Down42	-0.640553
	ENSECAG00000007112	ENSECAG00000007112	Down41	-0.831601					Down40	-0.652355
	ENSECAG00000012059	OPLAH	Down40	-0.838903					Down35	-0.670492
	ENSECAG00000023782	SLC16A13	Down10	-1.20782					Down2	-1.18221

Group 7	ENSECAG00000011439	DHX58			Up4	4.61608	Up4	3.09973	Up8	3.19554
	ENSECAG00000009020	IFI44			Up5	4.44821	Up6	2.17917	Up7	3.27331
	ENSECAG00000009618	IFI44L			Up8	3.82991	Up7	1.77632	Up10	2.80855
	ENSECAG00000001324	ISG15			Up9	3.01778	Up8	1.74358	Up11	2.04705
	ENSECAG00000000284	ENSECAG00000000284			Up10	2.99697	Up11	1.43109	Up16	1.72675
	ENSECAG00000011776	MX1			Up11	2.92668	Up12	1.40675	Up12	2.01323
	ENSECAG00000012132	ENSECAG00000012132			Up19	2.21372	Up20	1.26429	Up24	1.23645
	ENSECAG00000014422	OAS2			Up20	2.11659	Up50	0.897975	Up25	1.21193
	ENSECAG00000024429	IRF9			Up23	1.95478	Up10	1.533	Up21	1.36893
	ENSECAG00000001598	IFI35			Up25	1.56816	Up70	0.806505	Up29	1.12405
	ENSECAG00000014226	ENSECAG00000014226			Up28	1.40464	Up30	1.10198	Up28	1.13741
	ENSECAG00000025265	7SK			Up30	1.23285	Up16	1.32949	Up17	1.63899
	ENSECAG00000021133	FDFT1			Down108	-0.603939	Down29	-0.745322	Down31	-0.68223
	ENSECAG00000023067	MVK			Down57	-0.717767	Down26	-0.753929	Down28	-0.697242
	ENSECAG00000016389	ACSS2			Down52	-0.729119	Down23	-0.791528	Down43	-0.636744
	ENSECAG00000019932	ENSECAG00000019932			Up17	2.33603	Up9	1.62176		
	ENSECAG00000009543	EPSTI1			Up31	1.21872	Up23	1.25582		
	ENSECAG00000014295	PI3			Up40	1.08421	Up44	0.920787		
	ENSECAG00000012662	SEMA3A			Down113	-0.596351	Down30	-0.741631		
	ENSECAG00000014601	GADD45A			Down111	-0.597874	Down42	-0.678195		
	ENSECAG00000010554	MSMO1			Down79	-0.666701	Down3	-1.18586		
	ENSECAG00000011271	IGFBP4			Down74	-0.670052	Down43	-0.673379		
	ENSECAG00000024167	DDX60			Up3	4.63181			Up6	3.31012
	ENSECAG00000007881	IFIH1			Up6	4.40685			Up4	3.5855
	ENSECAG00000010185	ENSECAG00000010185			Up16	2.33874			Up14	1.90592
	ENSECAG00000025328	ENSECAG00000025328			Up18	2.28563			Up13	1.90771
	ENSECAG00000000968	ENSECAG00000000968			Up21	2.10439			Up33	1.0504
	ENSECAG00000027056	ENSECAG00000027056			Up22	1.95999			Up19	1.51838
	ENSECAG00000001481	SAMD9L			Up27	1.48532			Up40	0.876975
	ENSECAG00000021220	ENSECAG00000021220			Up29	1.34581			Up46	0.789085
	ENSECAG00000012331	PARP9			Up38	1.12343			Up60	0.692215
	ENSECAG00000011726	EIF2AK2			Up41	1.04597			Up47	0.781161
	ENSECAG00000001399	SAMD9			Up51	0.816776			Up95	0.600358
	ENSECAG00000010055	RSAD2			Up52	0.780623			Up51	0.730225
	ENSECAG00000002264	ISL1			Down117	-0.593754			Down38	-0.664083
	ENSECAG00000010566	ENSECAG00000010566			Down65	-0.699738			Down50	-0.608559
	ENSECAG00000019557	LDLR			Down58	-0.712427			Down26	-0.715306
	ENSECAG00000011206	MVD			Down40	-0.779099			Down20	-0.736368
	ENSECAG00000015180	NEXN					Up21	1.26305	Up58	0.702545
	ENSECAG00000016232	IL22RA1					Up91	0.728298	Up48	0.774814
	ENSECAG00000008852	ABCA1					Up160	0.603089	Up53	0.717703
	ENSECAG00000010720	ELOVL6					Down28	-0.752294	Down17	-0.747894
	ENSECAG00000014658	SCD					Down17	-0.898684	Down55	-0.590867
	ENSECAG00000005106	FIBIN					Down15	-0.917468	Down18	-0.740236
	ENSECAG00000008637	HMGCS1					Down14	-0.935547	Down24	-0.72822
	ENSECAG00000015140	LIPG					Down12	-0.942586	Down7	-0.989717
Group 8	ENSECAG00000000714	FAM26D	Up1	inf						
	ENSECAG00000022734	SGPP2	Up4	inf						
	ENSECAG00000026877	C17orf98	Up5	inf						
	ENSECAG00000009955	TEX43	Up6	inf						
	ENSECAG00000027680	ENSECAG00000027680	Up7	inf						
	ENSECAG00000007414	SV2A	Up9	4.7725						
	ENSECAG00000000207	ACTA1	Up10	4.27728						
	ENSECAG00000009535	FCER1G	Up11	3.62833						
	ENSECAG00000024000	PYGM	Up12	3.57668						
	ENSECAG00000009793	TAGLN3	Up14	3.0704						
	ENSECAG00000004408	GPR1	Up16	2.79532						
	ENSECAG00000008107	FERMT3	Up18	2.34706						
	ENSECAG00000009256	CPE	Up19	2.33724						
	ENSECAG00000019367	ENSECAG00000019367	Up20	2.03985						
	ENSECAG00000016226	ANKRD35	Up21	1.93647						
	ENSECAG00000012826	CDO1	Up22	1.93113						
	ENSECAG00000006922	ENSECAG00000006922	Up23	1.86167						
	ENSECAG00000015232	ZNF619	Up24	1.82611						
	ENSECAG00000008044	PPP1R27	Up25	1.76342						
	ENSECAG00000018052	ZNF514	Up27	1.59839						
	ENSECAG00000015717	TSSK3	Up29	1.54859						
	ENSECAG00000018029	THSD1	Up30	1.51379						
	ENSECAG00000012898	PNLDC1	Up34	1.39412						
	ENSECAG00000007194	CHRNE	Up36	1.3793						
	ENSECAG00000009142	ENSECAG00000009142	Up37	1.37497						
	ENSECAG00000020764	ENSECAG00000020764	Up38	1.34451						
	ENSECAG00000005115	ZNF454	Up42	1.2287						
	ENSECAG00000010656	HBEGF	Up43	1.22256						

	ENSECAG00000012746	STAR	Up45	1.16959
	ENSECAG00000020952	ENSECAG00000020952	Up48	1.13517
	ENSECAG00000008668	CSF3	Up49	1.11637
	ENSECAG00000012179	RGS2	Up50	1.1118
	ENSECAG00000024298	PTPN22	Up52	1.06985
	ENSECAG00000021582	ENSECAG00000021582	Up54	1.06426
	ENSECAG00000016648	IL5	Up55	1.05992
	ENSECAG00000008723	CASP12	Up56	1.04135
	ENSECAG00000013528	AVIL	Up59	1.01567
	ENSECAG00000018966	FBXO48	Up60	1.01195
	ENSECAG00000014572	ENSECAG00000014572	Up61	0.989668
	ENSECAG00000003681	ENSECAG00000003681	Up62	0.980483
	ENSECAG00000020923	LEAP2	Up63	0.980251
	ENSECAG00000016085	SYCP2	Up64	0.967566
	ENSECAG00000012653	DYRK4	Up65	0.965215
	ENSECAG00000012680	N4BP2L1	Up66	0.961023
	ENSECAG00000008533	ITGA10	Up68	0.951696
	ENSECAG00000024279	FAM200B	Up69	0.945443
	ENSECAG00000013527	ENSECAG00000013527	Up70	0.939975
	ENSECAG00000006567	SLITRK4	Up71	0.939637
	ENSECAG00000010606	CCDC169	Up73	0.930152
	ENSECAG00000023507	GPR19	Up74	0.925208
	ENSECAG00000013748	KRT25	Up76	0.921815
	ENSECAG00000003669	HLA-DMA	Up78	0.909888
	ENSECAG00000026852	ZCCHC10	Up79	0.905548
	ENSECAG00000005249	IQUB	Up81	0.883674
	ENSECAG00000012801	KBTBD3	Up83	0.868281
	ENSECAG00000010700	CREG2	Up84	0.844446
	ENSECAG00000021939	FAM122C	Up88	0.832568
	ENSECAG00000018428	HSPH1	Up89	0.831423
	ENSECAG00000002021	ENSECAG00000002021	Up90	0.829023
	ENSECAG00000011618	EDN1	Up92	0.824924
	ENSECAG00000001667	DEPDC7	Up93	0.823128
	ENSECAG00000002452	CCDC121	Up94	0.820488
	ENSECAG00000013653	TPMT	Up95	0.81773
	ENSECAG00000004698	PTGR1	Up97	0.816365
	ENSECAG00000011003	PDE4B	Up98	0.814737
	ENSECAG00000022568	TMEM154	Up99	0.809303
	ENSECAG00000019742	CCDC126	Up100	0.807428
	ENSECAG00000024185	GNG11	Up102	0.778049
	ENSECAG00000002759	MTRF1L	Up103	0.776082
	ENSECAG00000020892	TLR10	Up105	0.770745
	ENSECAG00000013766	E2F5	Up106	0.766488
	ENSECAG00000016965	SUV39H2	Up107	0.764009
	ENSECAG00000020223	HACD1	Up108	0.763083
	ENSECAG00000020380	COMMD8	Up109	0.761872
	ENSECAG00000004590	TIPIN	Up111	0.758445
	ENSECAG00000001407	CHSY3	Up112	0.756332
	ENSECAG00000013255	ENSECAG00000013255	Up113	0.755364
	ENSECAG00000006869	MAP9	Up117	0.748459
	ENSECAG00000013661	FAM184B	Up122	0.740297
	ENSECAG00000017348	BCAP29	Up123	0.736949
	ENSECAG00000011592	HNMT	Up124	0.734835
	ENSECAG00000012354	LMLN	Up125	0.725545
	ENSECAG00000015510	FGF7	Up126	0.723648
	ENSECAG00000014615	STX2	Up127	0.719371
	ENSECAG00000010953	TNIP3	Up128	0.717209
	ENSECAG00000019097	TANK	Up131	0.710888
	ENSECAG00000016351	ENSECAG00000016351	Up132	0.71022
	ENSECAG00000022765	ZC3H8	Up135	0.709287
	ENSECAG00000022541	ZNF25	Up137	0.706972
	ENSECAG00000007540	CYP7B1	Up138	0.706454
	ENSECAG00000010696	ENSECAG00000010696	Up139	0.705146
	ENSECAG00000016586	NUCB2	Up140	0.704499
	ENSECAG00000010433	CHMP2B	Up141	0.700278
	ENSECAG00000010663	SKIL	Up142	0.698517
	ENSECAG00000016844	MEIOC	Up143	0.696118
	ENSECAG00000009108	C1orf189	Up146	0.693171
	ENSECAG00000006803	SYTL2	Up147	0.69218
	ENSECAG00000024373	ZBTB11	Up148	0.686358
	ENSECAG00000016487	ATP6V1E1	Up149	0.68053
	ENSECAG00000010450	CCDC59	Up150	0.677447
	ENSECAG00000014597	MCTS1	Up152	0.675524
	ENSECAG00000019989	SNRPG	Up155	0.671256
	ENSECAG00000011295	THAP2	Up157	0.668933
	ENSECAG00000017572	ZNF280C	Up158	0.667444

	ENSECAG00000024614	FAM161A	Up159	0.66698			
	ENSECAG00000003510	TXNDC9	Up160	0.666237			
	ENSECAG00000024815	RFESD	Up163	0.663531			
	ENSECAG00000008493	SERPINB10	Up164	0.662478			
	ENSECAG00000004919	CACYBP	Up165	0.659535			
	ENSECAG00000026897	MRPS36	Up166	0.655053			
	ENSECAG00000008428	CEP83	Up167	0.654762			
	ENSECAG00000023743	CCDC58	Up168	0.654648			
	ENSECAG00000002943	ENSECAG00000002943	Up169	0.654099			
	ENSECAG00000024870	CWH43	Up171	0.653246			
	ENSECAG00000024704	CETN3	Up172	0.653123			
	ENSECAG00000000880	SPATA33	Up173	0.652458			
	ENSECAG00000008390	FOXN2	Up174	0.651412			
	ENSECAG00000010323	ENSECAG00000010323	Up175	0.651347			
	ENSECAG00000016530	LYPLA1	Up176	0.64999			
	ENSECAG00000009675	N4BP2	Up178	0.649494			
	ENSECAG00000009898	UEVLD	Up179	0.649393			
	ENSECAG00000015185	AK6	Up180	0.649205			
	ENSECAG00000000546	CCDC62	Up181	0.648597			
	ENSECAG00000009776	CEP57L1	Up182	0.646206			
	ENSECAG00000024399	FGF12	Up183	0.645883			
	ENSECAG00000021026	BCAS2	Up185	0.645444			
	ENSECAG00000002513	ERCC6L	Up186	0.642977			
	ENSECAG00000013569	LAMTOR3	Up187	0.642613			
	ENSECAG00000015459	ENSECAG00000015459	Up188	0.642552			
	ENSECAG00000015237	PIBF1	Up189	0.642502			
	ENSECAG00000015355	CLK1	Up192	0.640763			
	ENSECAG00000018554	ENSECAG00000018554	Up194	0.639487			
	ENSECAG00000011862	C11orf1	Up195	0.638822			
	ENSECAG00000015376	GLRX2	Up198	0.631247			
	ENSECAG00000015464	RPL36AL	Up201	0.626847			
	ENSECAG00000005764	ENSECAG00000005764	Up203	0.625927			
	ENSECAG00000005178	CCSAP	Up205	0.624865			
	ENSECAG00000023869	CRLS1	Up206	0.624073			
	ENSECAG00000015765	THAP6	Up207	0.621924			
	ENSECAG00000004316	HRSP12	Up208	0.621403			
	ENSECAG00000019506	ARRDC3	Up209	0.620906			
	ENSECAG00000019146	NDUFAF6	Up210	0.620436			
	ENSECAG00000006587	RWDD1	Up211	0.620345			
	ENSECAG00000008716	EIF3J	Up213	0.61719			
	ENSECAG00000015757	RPL26	Up214	0.616644			
	ENSECAG00000018478	NIFK	Up216	0.614659			
	ENSECAG00000011393	GKAP1	Up217	0.614196			
	ENSECAG00000018693	ENSECAG00000018693	Up218	0.612525			
	ENSECAG00000011218	HAT1	Up219	0.610503			
	ENSECAG00000017063	TIMM9	Up220	0.610056			
	ENSECAG00000014668	ZNF614	Up222	0.608307			
	ENSECAG00000009780	UQCRB	Up223	0.607478			
	ENSECAG00000012316	DTWD1	Up224	0.605808			
	ENSECAG00000007803	LSM3	Up226	0.602689			
	ENSECAG00000018635	ZNF674	Up227	0.601725			
	ENSECAG00000010661	RPL21	Up228	0.601547			
	ENSECAG00000017850	TAF1D	Up229	0.601284			
	ENSECAG00000016758	ENSECAG00000016758	Up230	0.601139			
	ENSECAG00000019035	LIPT1	Up231	0.60087			
	ENSECAG00000008524	SSBIP1	Up233	0.598617			
	ENSECAG00000015468	MGAT4A	Up234	0.598338			
	ENSECAG00000015373	ENSECAG00000015373	Up235	0.593591			
	ENSECAG00000020566	DYNC2LI1	Up238	0.589788			
	ENSECAG00000011795	OSGEPL1	Up239	0.589122			
	ENSECAG00000024976	OCIAD1	Up240	0.588753			
	ENSECAG00000017059	FAM105A	Up241	0.587727			
	ENSECAG00000014972	PHF11	Up242	0.587703			
	ENSECAG00000009760	GMNN	Up245	0.585393			
	ENSECAG00000012399	SUB1	Up246	0.583617			
	ENSECAG00000014530	SEPT10	Up247	0.581098			
	ENSECAG00000013324	TXN	Up248	0.58067			
	ENSECAG00000023217	KLHL17	Down181	-0.580187			
	ENSECAG00000011637	SLC1A5	Down180	-0.580647			
	ENSECAG00000017955	SLC12A9	Down179	-0.580741			
	ENSECAG00000000402	NDUFS7	Down178	-0.580877			
	ENSECAG00000022744	RHBDD3	Down177	-0.582825			
	ENSECAG00000014263	ENSECAG00000014263	Down176	-0.585909			
	ENSECAG00000002668	RHOB	Down174	-0.586719			
	ENSECAG00000011565	GMPPA	Down173	-0.587266			
	ENSECAG00000008220	ENSECAG00000008220	Down172	-0.587499			

	ENSECAG00000012351	FUK	Down171	-0.588028				
	ENSECAG00000009148	LPAR2	Down169	-0.589867				
	ENSECAG00000022309	DGKQ	Down168	-0.590556				
	ENSECAG00000023189	DDAH2	Down167	-0.593593				
	ENSECAG00000003398	MARCKS	Down166	-0.593864				
	ENSECAG00000024775	PRKD2	Down165	-0.594245				
	ENSECAG00000008659	SHISA5	Down164	-0.594512				
	ENSECAG00000016677	MFSD3	Down163	-0.597456				
	ENSECAG00000019788	WDR83	Down162	-0.598005				
	ENSECAG00000026863	EFS	Down161	-0.59856				
	ENSECAG00000012961	HSPG2	Down159	-0.600732				
	ENSECAG00000014491	NLRC5	Down158	-0.602386				
	ENSECAG00000008298	WNT10B	Down154	-0.605397				
	ENSECAG00000023401	ZNF414	Down153	-0.60616				
	ENSECAG00000021488	DYRK1B	Down152	-0.606423				
	ENSECAG00000018474	DDR1	Down151	-0.607061				
	ENSECAG00000021964	ASB9	Down150	-0.607786				
	ENSECAG00000012006	BCL9L	Down149	-0.608293				
	ENSECAG00000023274	LAMA5	Down148	-0.60959				
	ENSECAG00000021765	TSPO	Down145	-0.614029				
	ENSECAG00000017916	ENSECAG00000017916	Down144	-0.614305				
	ENSECAG00000017838	IGFLR1	Down143	-0.615358				
	ENSECAG00000015936	ENSECAG00000015936	Down142	-0.615914				
	ENSECAG00000012571	ENSECAG00000012571	Down140	-0.618863				
	ENSECAG00000020484	TBCC	Down139	-0.621186				
	ENSECAG00000021290	MBD6	Down136	-0.625814				
	ENSECAG00000002876	FAAP100	Down133	-0.629622				
	ENSECAG00000018245	EPHX3	Down132	-0.629753				
	ENSECAG00000017322	METTL12	Down131	-0.63042				
	ENSECAG00000012500	AP5Z1	Down129	-0.637977				
	ENSECAG00000017084	DPYSL4	Down128	-0.638735				
	ENSECAG00000016798	MIEF2	Down127	-0.64028				
	ENSECAG00000008910	KLRG2	Down126	-0.640342				
	ENSECAG00000011354	DHX34	Down125	-0.642514				
	ENSECAG00000026952	ENSECAG00000026952	Down123	-0.64348				
	ENSECAG00000009314	TMEM132A	Down122	-0.643783				
	ENSECAG00000010992	NFATC4	Down121	-0.644947				
	ENSECAG00000008387	TSC22D4	Down116	-0.650186				
	ENSECAG00000009361	COL5A1	Down115	-0.650422				
	ENSECAG00000005577	GPR39	Down114	-0.651523				
	ENSECAG00000015448	TAPBP	Down112	-0.653639				
	ENSECAG00000001072	TYMP	Down109	-0.657227				
	ENSECAG00000021314	COL4A2	Down108	-0.659806				
	ENSECAG00000004662	SPTSSA	Down107	-0.660094				
	ENSECAG00000017905	LTBP4	Down105	-0.661668				
	ENSECAG00000002142	TRIP6	Down102	-0.66844				
	ENSECAG00000022526	CDH15	Down101	-0.669839				
	ENSECAG00000016695	SLC27A5	Down99	-0.678787				
	ENSECAG00000020085	RELL2	Down98	-0.681381				
	ENSECAG00000007615	PTRH1	Down95	-0.683101				
	ENSECAG00000012759	PLPPR2	Down94	-0.686553				
	ENSECAG00000020780	PELP1	Down92	-0.69176				
	ENSECAG00000018161	C1QTNF5	Down91	-0.692114				
	ENSECAG00000024412	ARHGAP39	Down90	-0.696513				
	ENSECAG00000021280	SHISA4	Down89	-0.697033				
	ENSECAG00000015814	LRRC73	Down87	-0.698787				
	ENSECAG00000015240	PROM2	Down85	-0.702809				
	ENSECAG00000006999	PLEKHG4	Down84	-0.702886				
	ENSECAG00000009891	ARMC5	Down83	-0.703847				
	ENSECAG00000018111	NAPRT	Down82	-0.704411				
	ENSECAG00000021034	YDJC	Down78	-0.708416				
	ENSECAG00000019381	MEGF8	Down77	-0.713517				
	ENSECAG00000013608	KAZALD1	Down76	-0.714613				
	ENSECAG00000013419	MNT	Down73	-0.721419				
	ENSECAG00000014566	WISP2	Down71	-0.723337				
	ENSECAG00000023946	SDC3	Down70	-0.72685				
	ENSECAG00000011353	MFSD7	Down68	-0.730068				
	ENSECAG00000006116	PPP1R35	Down67	-0.73076				
	ENSECAG00000014470	EEPD1	Down66	-0.731131				
	ENSECAG00000004551	FOXG1	Down64	-0.741996				
	ENSECAG00000011160	TP53INP2	Down63	-0.747131				
	ENSECAG00000017501	TOR4A	Down62	-0.747398				
	ENSECAG00000006619	ZNF688	Down61	-0.751039				
	ENSECAG00000013524	ZNF219	Down60	-0.751192				
	ENSECAG00000011907	C19orf54	Down59	-0.751823				
	ENSECAG00000002911	KCTD11	Down58	-0.75277				

	ENSECAG00000017451	FAM110A	Down56 -0.770603			
	ENSECAG00000015612	FAM212A	Down55 -0.785398			
	ENSECAG00000007474	SPHK2	Down54 -0.791489			
	ENSECAG00000002061	NEURL2	Down53 -0.792894			
	ENSECAG00000006058	FUT1	Down49 -0.803043			
	ENSECAG00000000440	TMEM8B	Down48 -0.803244			
	ENSECAG00000024202	XKR8	Down46 -0.804649			
	ENSECAG00000018426	ERF	Down45 -0.812761			
	ENSECAG00000023399	GADD45G	Down37 -0.848082			
	ENSECAG00000022258	SDSL	Down35 -0.85835			
	ENSECAG00000006591	ATG9B	Down32 -0.873			
	ENSECAG00000002570	HS3ST1	Down31 -0.884388			
	ENSECAG00000007647	COL5A3	Down30 -0.89889			
	ENSECAG00000006133	NXPH4	Down28 -0.90606			
	ENSECAG00000013867	ENSECAG00000013867	Down24 -0.96001			
	ENSECAG00000006208	MBLAC1	Down23 -0.976957			
	ENSECAG00000012116	CARNS1	Down22 -0.97915			
	ENSECAG00000015692	SERTAD4	Down19 -1.02147			
	ENSECAG00000024458	KCTD7	Down18 -1.04533			
	ENSECAG00000011161	ENSECAG00000011161	Down16 -1.06533			
	ENSECAG00000026812	FANCF	Down11 -1.18548			
	ENSECAG00000007684	SLC2A4	Down9 -1.22492			
	ENSECAG00000009912	PCSK1N	Down7 -1.32892			
	ENSECAG00000018591	CTU1	Down6 -1.35574			
	ENSECAG00000025020	FHL1	Down5 -1.35746			
	ENSECAG00000009027	WBSCR27	Down4 -1.4755			
	ENSECAG00000014282	NPPB	Down3 -1.81488			
	ENSECAG00000003656	NANOS1	Down1 -inf			
Group 9	ENSECAG00000015034	PLAC8		Up1 inf		
	ENSECAG00000019309	GBP2		Up13 2.77326		
	ENSECAG00000004306	ENSECAG00000004306		Up24 1.70432		
	ENSECAG00000008809	OAS3		Up26 1.56128		
	ENSECAG00000010036	SLC15A3		Up32 1.21219		
	ENSECAG00000016217	RNF213		Up34 1.18669		
	ENSECAG00000009686	DTX3L		Up36 1.1458		
	ENSECAG00000019411	HERC6		Up39 1.0866		
	ENSECAG00000002176	ENSECAG00000002176		Up42 0.996487		
	ENSECAG00000017394	ZC3HAV1		Up44 0.95748		
	ENSECAG00000020562	AXDND1		Up45 0.929863		
	ENSECAG00000013435	OAS1		Up46 0.908969		
	ENSECAG00000021989	DDX58		Up47 0.896421		
	ENSECAG00000015395	HERC5		Up48 0.889434		
	ENSECAG00000012773	CMPK2		Up50 0.862742		
	ENSECAG00000009937	UBE2N		Up54 0.754702		
	ENSECAG00000001514	ENSECAG00000001514		Up55 0.750024		
	ENSECAG00000022601	PARP12		Up59 0.679275		
	ENSECAG00000020384	ENSECAG00000020384		Up60 0.638286		
	ENSECAG00000006930	ENSECAG00000006930		Up61 0.637825		
	ENSECAG00000023195	ENSECAG00000023195		Up62 0.631552		
	ENSECAG00000004349	IFIT5		Up63 0.621803		
	ENSECAG00000001560	SCO2		Down128 -0.580853		
	ENSECAG00000016361	COL18A1		Down127 -0.58119		
	ENSECAG00000013728	MFSD2A		Down126 -0.583615		
	ENSECAG00000010237	FBXL6		Down124 -0.586447		
	ENSECAG00000001342	SCAND1		Down123 -0.588235		
	ENSECAG00000005017	FBXW9		Down122 -0.590475		
	ENSECAG00000022018	TRIM47		Down116 -0.593891		
	ENSECAG00000017795	ENSECAG00000017795		Down114 -0.595276		
	ENSECAG00000010553	NGF		Down112 -0.596973		
	ENSECAG00000018835	FAM46B		Down110 -0.601717		
	ENSECAG00000008333	MSC		Down109 -0.601983		
	ENSECAG00000003816	JUNB		Down107 -0.6043		
	ENSECAG00000002992	MID1IP1		Down106 -0.604792		
	ENSECAG00000001265	TMEM223		Down105 -0.60591		
	ENSECAG00000004050	ENSECAG00000004050		Down102 -0.610559		
	ENSECAG00000000138	ACVR2A		Down96 -0.620891		
	ENSECAG00000024319	ENSECAG00000024319		Down92 -0.634334		
	ENSECAG00000019789	STOML1		Down91 -0.635372		
	ENSECAG00000006931	ATF4		Down90 -0.636222		
	ENSECAG00000004791	TICAM1		Down88 -0.638507		
	ENSECAG00000011779	ARID3A		Down87 -0.639476		
	ENSECAG00000013794	MRPS12		Down83 -0.649639		
	ENSECAG00000014783	ENSECAG00000014783		Down78 -0.668662		
	ENSECAG00000021308	APBA3		Down77 -0.669118		
	ENSECAG00000016405	REPIN1		Down76 -0.669615		

	ENSECAG00000011011	SNAPC2		Down75	-0.669702			
	ENSECAG00000024720	ENTHD2		Down73	-0.68113			
	ENSECAG00000016486	ARRDC2		Down72	-0.682995			
	ENSECAG00000017157	IER3		Down71	-0.685588			
	ENSECAG00000020178	WNT9A		Down69	-0.691772			
	ENSECAG00000012599	TRMT61A		Down68	-0.694494			
	ENSECAG00000024986	ENSECAG00000024986		Down66	-0.697917			
	ENSECAG00000004492	RRS1		Down64	-0.701231			
	ENSECAG00000000529	MAP1S		Down62	-0.709554			
	ENSECAG00000022294	ENSECAG00000022294		Down60	-0.711121			
	ENSECAG00000016441	DOHH		Down59	-0.711948			
	ENSECAG00000008194	MIDN		Down55	-0.724055			
	ENSECAG00000014794	MIIP		Down51	-0.729532			
	ENSECAG00000015758	IRX2		Down50	-0.730415			
	ENSECAG00000013124	BCL3		Down49	-0.730821			
	ENSECAG00000016942	NUDT14		Down48	-0.731937			
	ENSECAG00000013787	HMX3		Down45	-0.747514			
	ENSECAG00000021909	C14orf80		Down44	-0.751221			
	ENSECAG00000019359	DUS3L		Down42	-0.768069			
	ENSECAG00000010803	ENSECAG00000010803		Down41	-0.778312			
	ENSECAG00000026965	SOCS1		Down36	-0.789464			
	ENSECAG00000021887	TRIM62		Down35	-0.803212			
	ENSECAG00000022195	ENSECAG00000022195		Down34	-0.804666			
	ENSECAG00000004504	C19orf52		Down33	-0.810494			
	ENSECAG00000022697	TRABD2B		Down28	-0.843641			
	ENSECAG00000013721	SH2D5		Down26	-0.854379			
	ENSECAG00000024586	ENSECAG00000024586		Down25	-0.85534			
	ENSECAG00000015715	ADAMTS15		Down24	-0.861468			
	ENSECAG00000014511	IL11		Down16	-0.96058			
	ENSECAG00000012197	SPATA2L		Down8	-1.10335			
	ENSECAG00000018561	CDKN2B		Down7	-1.22934			
	ENSECAG00000022402	GFOD1		Down6	-1.23897			
	ENSECAG00000014921	GPRC5A		Down4	-1.33756			
	ENSECAG00000003025	HLX		Down2	-1.63124			
Group 10	ENSECAG00000003585	RPL19				Up17	1.32346	
	ENSECAG00000024426	PDAP1				Up18	1.29205	
	ENSECAG00000002024	ENSECAG00000002024				Up24	1.24544	
	ENSECAG00000006844	CALD1				Up25	1.19409	
	ENSECAG00000006493	PTMA				Up26	1.15249	
	ENSECAG00000019300	CARD9				Up28	1.12444	
	ENSECAG00000008785	ENSECAG00000008785				Up31	1.09935	
	ENSECAG00000016025	SNRNP27				Up32	1.07142	
	ENSECAG00000008257	RBMX2				Up35	1.00488	
	ENSECAG00000011155	ATP6V1G1				Up36	0.995159	
	ENSECAG00000005090	BASP1				Up39	0.962889	
	ENSECAG00000009472	CLSPN				Up40	0.955786	
	ENSECAG00000014983	HIRIP3				Up41	0.951502	
	ENSECAG00000008992	HYPK				Up42	0.944431	
	ENSECAG00000021583	LMOD1				Up46	0.911345	
	ENSECAG00000008506	HMMR				Up48	0.903499	
	ENSECAG00000010638	SREK1IP1				Up49	0.900818	
	ENSECAG00000014412	ZMAT2				Up51	0.897111	
	ENSECAG00000009621	INCENP				Up52	0.887348	
	ENSECAG00000009886	TRAF3IP1				Up54	0.874669	
	ENSECAG00000010132	HMGB2				Up55	0.874481	
	ENSECAG00000022843	ZC3H13				Up57	0.851282	
	ENSECAG00000012666	PSIP1				Up58	0.842963	
	ENSECAG00000019845	SPAG7				Up59	0.838775	
	ENSECAG00000022972	TCHP				Up60	0.838251	
	ENSECAG00000008385	ENSECAG00000008385				Up63	0.828334	
	ENSECAG00000015871	CDCA8				Up64	0.821569	
	ENSECAG00000008649	CCDC12				Up65	0.821442	
	ENSECAG00000000734	HMGA1				Up66	0.812707	
	ENSECAG00000012708	DNAJC8				Up67	0.809498	
	ENSECAG00000012364	MYEOV2				Up68	0.808633	
	ENSECAG00000026810	FAM133B				Up69	0.806944	
	ENSECAG00000009987	PCNP				Up71	0.797147	
	ENSECAG00000017165	ENSA				Up72	0.797126	
	ENSECAG00000012760	LUC7L3				Up73	0.792121	
	ENSECAG00000010635	GPS2				Up74	0.784306	
	ENSECAG00000021311	NUDC				Up75	0.777538	
	ENSECAG00000026980	ENSECAG00000026980				Up76	0.774947	
	ENSECAG00000008928	ENSECAG00000008928				Up77	0.769984	
	ENSECAG00000024127	CENPF				Up78	0.767092	
	ENSECAG00000003089	ENSECAG00000003089				Up79	0.764132	

	ENSECAG00000023366	SUDS3			Up80	0.761729	
	ENSECAG00000014880	CFAP45			Up81	0.752188	
	ENSECAG00000003072	C8orf4			Up82	0.746632	
	ENSECAG00000023594	FAM50A			Up83	0.737634	
	ENSECAG00000026981	SRRM1			Up84	0.735645	
	ENSECAG00000000593	PTN			Up85	0.733931	
	ENSECAG00000013485	MFAP1			Up86	0.731433	
	ENSECAG00000016693	GOLIM4			Up89	0.729457	
	ENSECAG00000003766	GADD45GIP1			Up92	0.727814	
	ENSECAG00000013395	RBM25			Up93	0.72709	
	ENSECAG00000014949	TPM1			Up94	0.726056	
	ENSECAG00000003616	ENSECAG00000003616			Up95	0.725223	
	ENSECAG00000017015	MYH11			Up96	0.723533	
	ENSECAG00000024809	C1orf131			Up97	0.718484	
	ENSECAG00000023288	RNPS1			Up98	0.717031	
	ENSECAG00000010941	CCDC150			Up99	0.713529	
	ENSECAG00000006666	MURC			Up101	0.712238	
	ENSECAG00000026989	PDCD7			Up102	0.711159	
	ENSECAG00000008098	TCF7			Up103	0.707057	
	ENSECAG00000019569	ENSECAG00000019569			Up104	0.707021	
	ENSECAG00000014847	NUPR1			Up105	0.706403	
	ENSECAG00000012794	LYAR			Up106	0.705285	
	ENSECAG00000014812	KRT23			Up107	0.697669	
	ENSECAG00000013815	ENSECAG00000013815			Up108	0.694679	
	ENSECAG00000007123	ENSECAG00000007123			Up109	0.68695	
	ENSECAG00000019621	ENSECAG00000019621			Up110	0.686873	
	ENSECAG00000016947	UPF3B			Up112	0.680315	
	ENSECAG00000014069	GOLM1			Up113	0.679488	
	ENSECAG00000022323	PSMC3IP			Up114	0.679355	
	ENSECAG00000021350	SRSF4			Up115	0.676075	
	ENSECAG00000017963	E2F7			Up116	0.675926	
	ENSECAG00000021271	ENSECAG00000021271			Up117	0.67362	
	ENSECAG00000021276	BRD3			Up118	0.672743	
	ENSECAG00000018958	C9orf78			Up120	0.669381	
	ENSECAG00000010862	TNIP1			Up121	0.668438	
	ENSECAG00000005865	HAUS8			Up122	0.666204	
	ENSECAG00000011826	ATPIF1			Up123	0.666041	
	ENSECAG00000010367	RAD51AP1			Up124	0.665138	
	ENSECAG00000012231	DEK			Up125	0.663819	
	ENSECAG00000021097	ACIN1			Up126	0.660863	
	ENSECAG00000011870	PYM1			Up127	0.656765	
	ENSECAG00000013512	ENSECAG00000013512			Up128	0.655647	
	ENSECAG00000014953	KIF15			Up129	0.653919	
	ENSECAG00000011337	ODF2			Up130	0.652646	
	ENSECAG00000017141	UPF3A			Up131	0.646773	
	ENSECAG00000014242	MAD1L1			Up132	0.646476	
	ENSECAG00000016495	NUSAP1			Up133	0.646448	
	ENSECAG00000026853	CEP152			Up134	0.644139	
	ENSECAG00000018476	TPX2			Up136	0.642373	
	ENSECAG00000022315	BIN3			Up137	0.641456	
	ENSECAG00000002115	ENSECAG00000002115			Up139	0.639271	
	ENSECAG00000008466	ENSECAG00000008466			Up140	0.638539	
	ENSECAG00000026905	RPL37			Up141	0.637897	
	ENSECAG00000018538	PPIG			Up142	0.637641	
	ENSECAG00000003357	ING1			Up143	0.635161	
	ENSECAG00000012624	POLD3			Up144	0.634971	
	ENSECAG00000024902	ENSECAG00000024902			Up145	0.634489	
	ENSECAG00000013696	SLU7			Up146	0.633604	
	ENSECAG00000001897	SUMO2			Up148	0.628834	
	ENSECAG00000015060	PRR11			Up149	0.627963	
	ENSECAG00000020610	NDUFB10			Up151	0.61959	
	ENSECAG00000015496	ENSECAG00000015496			Up152	0.616784	
	ENSECAG00000007487	ENSECAG00000007487			Up153	0.61607	
	ENSECAG00000021538	CKAP2L			Up154	0.613215	
	ENSECAG00000017105	SEC62			Up155	0.610318	
	ENSECAG00000004534	CCDC136			Up156	0.605553	
	ENSECAG00000017076	NSRP1			Up158	0.603928	
	ENSECAG00000012779	IK			Up159	0.603291	
	ENSECAG00000000633	ZWINT			Up161	0.600546	
	ENSECAG00000000644	MAP7D3			Up161	0.600546	
	ENSECAG00000013640	CEP85			Up162	0.60029	
	ENSECAG00000022449	MEAF6			Up163	0.599962	
	ENSECAG00000012637	LENG1			Up165	0.598714	
	ENSECAG00000018394	MESDC2			Up166	0.597826	
	ENSECAG00000022285	ENSECAG00000022285			Up167	0.597506	
	ENSECAG00000018438	CEP55			Up168	0.596321	

	ENSECAG00000006129	NUP62			Up169	0.595882		
	ENSECAG00000014320	PPL			Up170	0.595836		
	ENSECAG00000008538	SPECC1			Up171	0.593915		
	ENSECAG00000019321	TXLNA			Up172	0.590412		
	ENSECAG00000012023	ENSECAG00000012023			Up173	0.590344		
	ENSECAG00000017006	CHMP4A			Up174	0.588888		
	ENSECAG00000014009	TPM2			Up175	0.587612		
	ENSECAG00000005815	LLPH			Up176	0.58692		
	ENSECAG00000011537	C14orf119			Up177	0.586732		
	ENSECAG00000011778	C11orf58			Up178	0.585521		
	ENSECAG00000015878	AMOT			Down62	-0.583045		
	ENSECAG00000008577	PPP3CB			Down61	-0.58889		
	ENSECAG00000022342	PKIA			Down60	-0.590756		
	ENSECAG00000024974	RNF11			Down59	-0.592537		
	ENSECAG00000017012	PTEN			Down58	-0.601453		
	ENSECAG00000010628	PPP1R14B			Down57	-0.602554		
	ENSECAG00000016862	SLC25A33			Down56	-0.608907		
	ENSECAG00000023284	ENSECAG00000023284			Down55	-0.619739		
	ENSECAG00000003002	CD9			Down54	-0.624481		
	ENSECAG00000014232	NR4A2			Down53	-0.625517		
	ENSECAG00000011223	HGF			Down52	-0.6264		
	ENSECAG00000010571	AGL			Down51	-0.636853		
	ENSECAG00000014113	CCNG2			Down50	-0.64024		
	ENSECAG00000021307	TGDS			Down49	-0.650154		
	ENSECAG00000001318	UBL3			Down48	-0.652025		
	ENSECAG00000002481	ENSECAG00000002481			Down47	-0.655159		
	ENSECAG00000012577	HOXA10			Down46	-0.658534		
	ENSECAG00000008215	TSC22D3			Down45	-0.666727		
	ENSECAG00000010827	ENSECAG00000010827			Down44	-0.667793		
	ENSECAG00000015783	PTBP2			Down41	-0.686615		
	ENSECAG00000005757	ENSECAG00000005757			Down40	-0.690087		
	ENSECAG00000019834	CYP39A1			Down39	-0.695967		
	ENSECAG00000021330	HMGCR			Down38	-0.700786		
	ENSECAG00000012062	EFNA5			Down37	-0.708252		
	ENSECAG00000005163	SLC38A6			Down36	-0.712477		
	ENSECAG00000011841	FAM174A			Down35	-0.717274		
	ENSECAG00000009515	ATRNL1			Down34	-0.729997		
	ENSECAG00000016183	STARD4			Down33	-0.739402		
	ENSECAG00000008425	LURAP1L			Down31	-0.740235		
	ENSECAG00000017787	FLVCR1			Down27	-0.752844		
	ENSECAG00000020317	RAP2C			Down25	-0.76238		
	ENSECAG00000024992	HSD17B7			Down24	-0.772415		
	ENSECAG00000006455	CYP51A1			Down22	-0.803576		
	ENSECAG00000000046	FAM13C			Down21	-0.819509		
	ENSECAG00000008926	RNF139			Down20	-0.827204		
	ENSECAG00000013998	SQLE			Down18	-0.853104		
	ENSECAG00000018059	SC5D			Down16	-0.913739		
	ENSECAG00000002626	GPR12			Down10	-0.983593		
	ENSECAG00000007020	C14orf28			Down8	-1.07536		
	ENSECAG00000011527	CASD1			Down7	-1.1038		
	ENSECAG00000021607	TXNDC8			Down6	-1.12117		
	ENSECAG00000024444	CTNNA2			Down5	-1.1567		
	ENSECAG00000003658	ENSECAG00000003658			Down4	-1.16691		
	ENSECAG00000006174	ENSECAG00000006174			Down1	-1.46428		
Group 11	ENSECAG00000027451	ENSECAG00000027451					Up2	inf
	ENSECAG00000016985	IL36G					Up20	1.39083
	ENSECAG00000027624	ENSECAG00000027624					Up26	1.18142
	ENSECAG00000015449	IRF8					Up32	1.07107
	ENSECAG00000014398	ENSECAG00000014398					Up35	1.01109
	ENSECAG00000015389	SV2B					Up38	0.908518
	ENSECAG00000022387	RUNDC3B					Up43	0.82593
	ENSECAG00000024909	FBXL2					Up54	0.713893
	ENSECAG00000015959	MYBL1					Up55	0.705196
	ENSECAG00000009742	S100A12					Up57	0.704123
	ENSECAG00000024016	IL13RA2					Up61	0.68763
	ENSECAG00000011226	CENPQ					Up63	0.6774
	ENSECAG00000024391	CD58					Up65	0.667648
	ENSECAG00000011292	MSR1					Up70	0.653605
	ENSECAG00000009925	ARNTL2					Up71	0.648288
	ENSECAG00000023416	F3					Up73	0.64621
	ENSECAG00000019890	VRK1					Up74	0.635457
	ENSECAG00000020270	CCNE2					Up77	0.631894
	ENSECAG00000010871	TMEM126B					Up80	0.623039
	ENSECAG00000027692	COX2					Up85	0.611617
	ENSECAG00000022189	PFDN4					Up86	0.611601

	ENSECAG00000019398	TFPI2				Up88 0.60962
	ENSECAG00000019817	SNRPF				Up90 0.608546
	ENSECAG00000027681	ND2				Up97 0.597166
	ENSECAG00000020878	ENSECAG00000020878				Up101 0.581476
	ENSECAG00000017926	TROAP				Down57 -0.582251
	ENSECAG00000002482	PQLC1				Down56 -0.58856
	ENSECAG00000011939	CLIP2				Down53 -0.595107
	ENSECAG00000008012	CHST3				Down52 -0.598346
	ENSECAG00000007907	ENSECAG00000007907				Down46 -0.619994
	ENSECAG00000009352	OBSL1				Down45 -0.621182
	ENSECAG00000017294	SNTB1				Down44 -0.624512
	ENSECAG00000021037	FEZF2				Down41 -0.64712
	ENSECAG00000017142	SEZ6				Down39 -0.663987
	ENSECAG00000000744	TBC1D2				Down34 -0.673914
	ENSECAG00000026973	BCL11B				Down21 -0.736266
	ENSECAG00000014357	DLL1				Down19 -0.739284
	ENSECAG00000008871	ALDH1A3				Down16 -0.75701
	ENSECAG00000009779	SORCS2				Down15 -0.772107
	ENSECAG00000013825	NOD1				Down14 -0.79745
	ENSECAG00000017019	ARHGAP33				Down13 -0.810786
	ENSECAG00000011401	CATSPERD				Down12 -0.814234
	ENSECAG00000014632	CPLX2				Down10 -0.904945
	ENSECAG00000023032	AZIN2				Down6 -1.00068
	ENSECAG00000017175	REM1				Down4 -1.09741
	ENSECAG00000013783	ENSECAG00000013783				Down3 -1.14067
	ENSECAG00000026054	U6				Down1 -inf

	**O/03**	**O/03-K186E**	**O/03-230**	**O/03-K186E-230**
Up-regulated genes	248	65	179	101
Down-regulated genes	181	128	62	57
**Total DEGs**	**429**	**193**	**241**	**158**
